# Extrinsic Anisotropy of Two‐Phase Newtonian Aggregates: Fabric Characterization and Parameterization

**DOI:** 10.1029/2021JB022232

**Published:** 2021-10-29

**Authors:** Albert de Montserrat, Manuele Faccenda, Giorgio Pennacchioni

**Affiliations:** ^1^ Dipartimento di Geoscienze Università degli Studi di Padova Padova Italy

## Abstract

Rocks of the Earth's crust and mantle commonly consist of different minerals with contrasting mechanical properties. During progressive, high‐temperature (ductile) deformation, these rocks develop extrinsic mechanical anisotropy linked to strain partitioning between different minerals, amount of accumulated strain, and bulk strain geometry. Extrinsic anisotropy plays an important role in a wide range of geodynamic processes up to the scale of mantle convection. However, the evolution of grain‐ and rock‐scale fabrics causing this anisotropy cannot be directly simulated in large‐scale numerical simulations. For two‐phase aggregates–a good rheological approximation of most Earth's rocks–we propose a method to indirectly approximate the extrinsic viscous anisotropy by combining (a) 3D mechanical models of rock fabrics, and (b) analytical effective medium theories. Our results confirm that weak inclusions induce substantial weakening by forming a network of weak thin layers with limited lateral connectivity. Consequently, even when the inclusion phase is extremely weak, structural weakening is not larger than 30–60%, less than in previous estimates. On the other hand, the presence of strong inclusions does not have a profound impact on the effective strength of the aggregate, and lineated fabrics only develop at relatively low viscosity contrasts. When rigid inclusions become clogged, however, the aggregate viscosity can increase over the theoretical upper bound. We show that the modeled grain‐scale fabrics can be parameterized as a function of the bulk deformation and material phase properties and combined with analytical solutions to approximate the anisotropic viscous tensor.

## Key Points

- 3D modeling of strain‐induced fabrics in Newtonian two‐phase aggregates under simple shear deformation
- Evolution of extrinsic viscous anisotropy
- Parameterization of the anisotropic viscous tensor

## 1 Introduction

Earth's dynamics, up to the scale of plate tectonics and deep mantle convection, are associated with high‐temperature, viscous rock flow by crystal‐plastic processes. Viscous deformation of rocks with grain‐scale compositional (mineralogical) heterogeneities commonly results in the development of an anisotropic fabric (referred to as extrinsic anisotropy) that arises from the 3D shape preferred orientation (SPO) of mineralogically distinct domains. The presence of extrinsic anisotropy may significantly influence the material properties (seismic anisotropy (Backus, 1962; Faccenda et al., 2019; Gee & Jordan, 1988), and rock strength (Dabrowski et al., 2012; Thielmann et al., 2020)) and a wide range of geological processes up to the scale of global tectonics (e.g., folding (Kocher et al., 2006; Mühlhaus et al., 2002), lithospheric instabilities (Lev & Hager, 2008; Perry‐Houts & Karlstrom, 2019), and mantle convection (Ballmer et al., 2017; Girard et al., 2016; Honda, 1986)).

While a good characterization of extrinsic anisotropy is necessary to quantify its impact on geodynamic processes, a framework to predict the evolution of the grain‐scale rock fabrics as a function of regional or global scale 3D convection patterns does not yet exist. Previous numerical studies considered compositionally layered media with simplified rheology, and the extrinsic anisotropy has been estimated for a strain‐insensitive fabric by the Voigt and Reuss upper and lower bounds (Christensen, 1987; Lev & Hager, 2008; Mühlhaus et al., 2002; Perry‐Houts & Karlstrom, 2019). Recent lab experiments at low finite strain have revealed that the effective strength of composites is strongly related to the initial geometry of the weak phase inclusion (Girard et al., 2016), which tends to form a network of layers of strain localization as strain increases. Dabrowski et al. (2012) and Thielmann et al. (2020) numerically studied the strength evolution of 2D, two‐phase aggregates at larger deformation, reporting effective strength drops of about 80%. These 2D simulations likely overestimate the degree of weakening as they implicitly assume ideal lateral interconnection of the weak layers.

The goal of this paper is to combine numerical and semi‐analytical methods to predict 3D strain‐induced fabrics of two‐phase (inclusion‐matrix) aggregates (representative of a wide spectrum of geological materials) and associated extrinsic viscous anisotropy. First, numerical tools are employed to reproduce 3D fabrics under simple shear deformation to quantify the relationship between the extrinsic viscous anisotropy, amount of strain and volume ratio between inclusion and matrix. Then, we demonstrate that the anisotropy of a two‐phase composite is well‐predicted by semi‐analytical solutions based on the Differential Effective Medium (DEM) theory. Finally, we parameterize the resulting 3D fabrics as a function of strain and strength contrast between the coexisting phases. For an arbitrary deformation state, the fabric can then be approximated, and the associated anisotropic viscous tensor can be calculated by the DEM.

## 2 Methods

We employ a Finite Differences solver for the 3D Stokes equations (T. Gerya, 2019) as the main tool to study the fabric development and associated evolution of the bulk viscosity in two‐phase aggregates in simple shear. Numerical models are complemented with solutions derived from the DEM theory.

### 2.1 Numerical Methods

Deformation is described by the Stokes equations for incompressible viscous flow:

(1) $$\begin{array}{ccc} \nabla \cdot \tau -\nabla p=0 & & \end{array}$$

(2) $$\begin{array}{ccc} \nabla \cdot v=0 & & \end{array}$$

where ∇ is the nabla operator, ******τ****** is the deviatoric stress tensor*, p* is pressure, and ******v****** is velocity. Equations 1 and 2 are solved employing a finite differences scheme combined with a particle‐in‐cell method (T. V. Gerya & Yuen, 2003) in the 3D Cartesian space. Tri‐linear interpolation resulting in a weighted arithmetic mean is employed to map the viscosity back and forth between Eulerian nodes and Lagrangian particles. Throughout this paper, we use (a) bold upper case Latin and lower case Greek letters to denote fourth and second order tensors, respectively; (b) bold lower case Latin letters to denote vectors; and (c) regular symbols to denote scalar values.

For simplicity we adopt an isotropic Newtonian rheology for each material phase, so that the relationship between stress and strain is given by the linear constitutive equation $\tau =2\eta \dot{\varepsilon }$, where *η* is the shear viscosity and $\dot{\varepsilon }$ is the deviatoric strain rate tensor. This simplified rheology does not account for the effects of dislocation creep, brittle failure, pressure‐solution, surface tension or other mechanisms that could affect the deformational behavior of a natural rock. Nonetheless, a Newtonian rheology is expected to be representative of the deformation behavior at mantle conditions where diffusion creep dominates (Ranalli, 1995).

Stokes equation are non‐dimensionalized using the characteristic scales of viscosity *η*_*c*_, length *l*_*c*_, and time *t*_*c*_:

(3) $$\eta_{c}=\min\left(\eta^{i},\eta^{m}\right)\;l_{c}=L\;t_{c}=\frac{1}{{\dot{\varepsilon }}^{bg}}$$

where the superscripts *i* and *m* stand respectively for inclusion and matrix, *L* is the length of the cubic aggregate, and ${\dot{\varepsilon }}^{bg}$ is the background strain rate. The choice of *η*_*c*_ is purely out of convenience, so that both normalized viscosities are integers.

#### 2.1.1 Model Setup

For a wide range of *P*‐*T* conditions, rock‐forming polymineral aggregates can be approximated by a population of dispersed particles (inclusions) within a matrix of distinct rheology where, in the simplest case, the initial rock geometry is isotropic with no shape preferred orientation of inclusions. This geometry is a good proxy of the fabric of many magmatic rocks, including mantle and oceanic rocks. Assuming a pyrolitic or the more depleted harzburgitic composition, above 410 km depth mantle rocks are made mainly of olivine and pyroxene (respectively, 60:40 and 70:30, Stixrude & Lithgow‐Bertelloni, 2012). In the mantle transition zone this proportion remains roughly constant as olivine crystals transform into the high pressure polyhmorphs wadsleytie and ringwoodite, and pyroxene transforms into majoritic garnet. The lower mantle is mainly composed by bridgmanite and ferropericlase (80:20; ignoring minor presence of Ca‐perovskite). After eclogitization the oceanic crust is made by omphacitic pyroxene and pyrope garnet, plus minor quartz (10%). In the transition zone, basalts are made by garnet and 10% stishovite. In the lower mantle, the subducted crust is formed by a four‐phase aggregate with about similar volume fractions and unknown relative strength, such that it is not yet possible to predict potential grain‐scale fabrics.

A simplified, but representative dimensionless model for such a composite consists of spherical inclusions (here with equal radius *r* = 0.1) randomly distributed within a cubic matrix of unit volume. This geometry avoids the complex crystal‐like shapes of natural aggregates that pose a computational challenge for numerical codes, particularly at large inclusion‐matrix viscosity contrast, as the stiness matrix resulting from the Stokes equations becomes more ill‐posed. In 3D high‐resolution models, this causes a signicant slow‐down in the convergence rate of the iterative method (Geometric Multigrid Method, or GMG) used to solve the linear system of equations (see next section 2.1.2). In addition, spherical inclusions generally deform into pseudo‐ellipsoids, so that tracking and quantifying the evolution of their shape is easier than for irregular shapes. Aggregates of (Mg,Fe)SiO_3_ perovskite and ferropericlase, synthesized at uppermost lower mantle equivalent conditions (Yamazaki et al., 2009) consist of clusters of ferropericlase equant grains of comparable grain sizes and shapes resulting in near‐isotropic samples prior to deformation. To a first approximation, the crystal shape ferropericlase grains is reasonably well approximated by spheres within a (Mg,Fe)SiO_3_ matrix. In other geological scenarios, porphyroclasts‐matrix aggregates in mylonites and bubble‐bearing magmatic rocks are well approximated by composites containing pseudo‐spherical inclusions in many cases.

The domain of the model is spatially discretized in an immutable and regular grid of cubic cells with 245 × 245 × 277 nodes. To simulate simple shear a kinematic reference frame is set where: the *X*‐axis is the shear direction; the *Y*‐axis is the vorticity axis, and the X–Y plane is the shear plane orthogonal to Z. Two rigid plates with thickness 0.08 are added to the top and bottom of the aggregate domain where unitary velocities ***v*** ^top^ = (1, 0, 0) and ***v*** ^bottom^ = (−1, 0, 0) are prescribed, so that the domain of the model is $\Omega \in \left[0,1\right]\times \left[0,1\right]\times \left[0,1.16\right]$ Periodic boundary conditions are prescribed at the vertical faces of the domain.

We explore the development of fabrics at different viscosity contrasts between the inclusion and matrix phases by fixing the viscosity of the weak phase to 1 and varying the viscosity of the strong phase, so that $\eta^{\text{strong}\;\text{phase}}\in \left[10,\;10^{2},\;10^{3}\right]$. The viscosity contrast between both phases is then defined as Δ*η* = *η* ^*i*^/*η* ^*m*^. We further study the cases of composites with different volume fractions of the least abundant phase $\phi \in \left[10,\;20,\;30\right]\;\%$.

#### 2.1.2 Model Convergence

3D Stokes problems with large discontinuous viscosity contrasts, such as those presented here, result in a linear system of equations with millions of degrees of freedom (∼66.5 millions in the models presented here) and highly spatially heterogeneous coefficients within the discretized elliptic operator. The linearized Stokes equations are solved iteratively via GMG, with Gauss‐Seidel smoothing operating on every V‐cycle level. This solution scheme works well for models with low and moderate inclusion‐matrix viscosity contrast, producing well resolved flow solutions. However, at high viscosity contrast the GMG does not converge to a well‐resolved flow solution after small finite strains when inclusion aspect ratio is different than one. May et al. (2015) employed matrix‐free operators and a combination of GMG and Algebraic Multigrid Methods (AMG) to solve a sinker problem with viscosity contrasts of up to six orders of magnitude. However, the problem was solved only for instantaneous flow and with spherical sinkers. Although promising, such solution schemes are yet to be tested for model set‐ups and complex geometries comparable to the ones in this paper. Here we employ the following strategy: (a) at the first time step we employ the PARDISO direct solver (De Coninck et al., 2016; Verbosio et al., 2017; Kourounis et al., 2018) at some intermediate multigrid level to obtain the exact solution; (b) the solution is prolonged to the finest multigrid level; (c) multigrid V‐cycles are performed until the desired tolerance is reached; (d) when the solution starts to diverge, the solution from the previous time step (initial guess) is reloaded and the V‐cycles restarted with the pressure and velocity relaxation parameters halved. The latter step ensures a safer updating of the unknowns and a more stable, although slower, convergence toward a well‐resolved solution.

#### 2.1.3 Deformation History of the Inclusions

An isolated, spherical, isotropic inclusion suspended in a viscous matrix transforms into an ellipsoid (the inclusion finite strain ellipsoid, FSE) by homogeneous deformation of the matrix. In aggregates of spatially dense inclusions, particle interactions result in more complex deformed inclusion shapes. For these complex shapes the amount of accumulated strain is evaluated by computing the finite strain equivalent ellipsoid (FSEE), that is, the average FSE of the inclusions. The FSEE is given by eigenvalues *a*_*i*_ and eigenvectors *λ*_*i*_, with *i* = 1, 2, 3, of the left stretch tensor (e.g., Becker et al., 2003), which define the length and orientation, respectively, of the FSEE semi‐axes in the Cartesian space. The FSE is computed *a posteriori* in a set of selected Lagrangian particles sampling homogeneously the inclusions. We use the routines included in the software D‐Rex (Kaminski et al., 2004) to compute the velocity gradient tensor, and update the deformation gradient tensor and the FSE. Arithmetic averaging of the Lagrangian particle FSE sampling a given inclusion is used to compute its FSEE. D‐Rex does not inject particles when the cells of the discretized domain become empty, yielding artifacts on FSE(E) at large strain when weak inclusions are extremely stretched and/or heterogeneously deformed.

### 2.2 Analytical and Semi‐Analytical Solutions for Viscous Tensors of Multi‐Phase Aggregates

The Voigt and Reuss bounds define the upper and lower limits, respectively, for any bulk material property of composites with continuous fibers. For a given field or mechanical property Ψ, the Voigt and Reuss (e.g., Handy, 1990) bounds are given by:

(4) $$\Psi^{Voigt}=\sum_{i}^{n}\phi_{i}\eta_{i}\;\Psi^{\;Reuss}={\left(\sum_{i}^{n}\frac{\phi_{i}}{\eta_{i}}\right)}^{-1}$$

where *i* is a material phase and *n* the total number of material phases. In our case, Equation 4 predict the orthogonal and parallel viscosities with respect to the fabric of a laminar material, respectively. Natural multi‐phase rocks rarely display a perfect laminar fabric and the Voigt and Reuss limits fail to provide accurate estimates of the mechanical properties. However, these limits still represent the upper and lower bounds of the mechanical properties of an aggregate. To overcome these limitations, alternatives methods in the field of micro‐mechanics (Mura, 1987; Nemat‐Nasser & Hori, 2013; J. Qu & Cherkaoui, 2006) based on Eshelby's work Eshelby (1957, 1959) have been developed and applied to geology in recent years (e.g., Dabrowski et al., 2012; Jiang, 2014; Jiang & Bhandari, 2018; Yang et al., 2019). These methods however work under the assumption of ellipsoidal inclusions. Here we employ the DEM, which additionally assumes aligned inclusions, to draw comparisons with the rheology of 3D models and explore its capability to quantify the viscous tensor in aggregates with realistic rock‐like fabrics. The DEM was first introduced by Roscoe (1952) to calculate the viscosity of suspensions of rigid particles and has been widely employed (e.g., Boucher, 1976; Dabrowski et al., 2012; Mainprice, 1997). The DEM tensorial formulation was developed by McLaughlin (1977):

(5) $$\frac{d\eta^{D}}{d\phi }=\frac{1}{1-\phi }\left(\eta^{i}-\eta^{D}\right):A$$

where ***η*** ^*D*^ is the viscosity tensor of the aggregate and **A** is the inclusion shape‐dependent interaction fourth order tensor (e.g., Jiang, 2014; Mainprice, 1997):

(6) $$A={\left[J^{s}+S:\left({\left(\eta^{D}\right)}^{-1}:\eta^{i}-J^{s}\right)\right]}^{-1}$$

where the symmetric fourth order identity tensor is defined as $J_{ijkl}^{s}=\frac{1}{2}\left(J_{ijkl}+J_{jikl}\right)$, with *J*_*ijkl*_ = *δ*_*ik*_ *δ*_*jl*_ being the fourth order identity tensor and *δ*_*ij*_ the Kroenecker's delta. For a general viscous material, the Eshelby tensor **S** is given by:

(7) $$S=J^{s}:T:\eta^{D}$$

where **T** is the fourth order Green interaction tensor (Lebensohn et al., 1998):

(8) $$T=\frac{a_{1}a_{2}a_{3}}{4\pi }\int_{0}^{2\pi }\int_{0}^{\pi }\frac{\xi \xi^{T}{\left(A^{v}\right)}^{-1}}{{\langle a^{2},\xi^{2}\rangle }^{3/2}}\sin\phi d\phi d\theta$$

where ***ξ*** = [ sin(*θ*) cos(*ϕ*), sin(*θ*) sin(*ϕ*), cos(*θ*)]^*T*^, 〈⋅, ⋅〉 indicates inner product, and

(9) $$A^{v}=\left[\begin{array}{cc} A & \xi \\ \xi^{T} & 0 \end{array}\right]$$

with $A_{ij}=\eta_{ikjn}^{D}\xi_{k}\xi_{n}$. The ordinary differential Equation 5 is solved by iteratively increasing *ϕ* (taking *ϕ* ^0^ = 0) until reaching the desired volume fraction, and integrated employing a fourth order Runge‐Kutta scheme. In the first iteration, the viscous tensor of the aggregate is defined by the viscosity of the matrix, that is, ***η*** ^*D*^(*ϕ* = 0) = 2**J** ^*s*^ *η* ^*m*^. In Appendix B we discuss the numerical implementation of Equation 5 and the associated computational cost.

The main downside of the DEM is that all the inclusions are assumed of equal shape and orientation. However, stress and strain are not evenly distributed among all the inclusions during shear deformation due to inclusion interactions. This results in a heterogeneous distribution of inclusion shapes and orientations. Therefore, to compare the numerical results with the DEM, we average the FSE of all the inclusions to obtain the FSEE.

## 3 Evolution of Two‐Phase Aggregates in Simple Shear Deformation

We first describe the evolution of a single spherical inclusion with Δ*η* = 10^±1^ and dimensionless radius 0.2. Then, we describe the development of the fabric and anisotropic viscosity for multiple inclusions. The fabric is quantied by the aspect ratio of the FSEE principal axes and the inclination (*α*) of the FSEE *a*_1_‐axis with respect to the horizontal shear plane.

### 3.1 Two‐Phase Aggregate With a Single Inclusion

**Figure 1:**
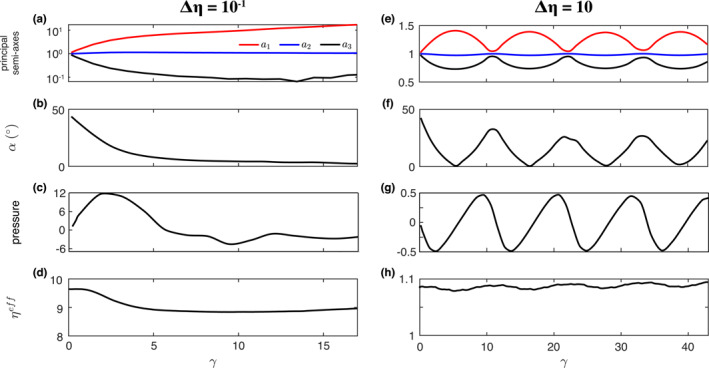
Evolution of the (a and e) principal semi‐axes of the FSE, (b and f) inclusion inclination (absolute value), (c and g) pressure at the center of the inclusion, and (d and h) effective shear viscosity evolution. Pressure is tracked at a single point located at the center of the matrix; small perturbations in the pressure inside the weak inclusion are related to the loss of resolution caused by severe flattening and stretching. The initial radius of the inclusion is 0.2.

During simple shear, a spherical weak inclusion transforms into an FSE with the maximum *a*_1_‐axis initially inclined at 45° with respect to the *X*‐axis. With increasing strain (a) the FSE *a*_1_ : *a*_3_ ratio increases with the intermediate *a*_2_‐axis (parallel to Y) rapidly growing initially of about 15% and then remaining constant in length for larger strain (Figure 1a); and (b) the maximum *a*_1_‐axis of the FSE progressively rotates in the XZ plane toward the *X*‐axis to achieve a nearly stable position at ca. *α* = 1.5° for *γ* > 15 (Figure 1b). As illustrated by 2D analytical solutions (Moulas et al., 2014), the pressure inside the inclusion depends on the viscosity contrast with the matrix and the maximum axis inclination *α*. In the case of a weak inclusion, the internal pressure increases to a maximum value at about *α* = 20°, and then decreases as the inclusion further rotates toward the Cartesian *X*‐axis. The pressure eventually becomes negative at *α* < 7°, and then remains roughly constant at higher strain (Figure 1c). The presence of a single weak inclusion weakens the bulk strength of the composite, inducing a decrease of the effective viscosity of about 10% with respect to the matrix viscosity *γ* > 5 (Figure 1d).

In simple shear, a single spherical inclusion stiffer than the matrix permanently rotates (Figure 1e and 1f). During a rotation cycle, the inclusion transforms into a 3‐axes ellipsoid having the *a*_1_‐and *a*_3_‐axis at their maximum and minimum, respectively, when the *a*_1_‐axis is aligned with the shear plane. As rotation continues, the inclusion recovers its spherical shape. As predicted by analytical expressions (Moulas et al., 2014), the pressure inside the inclusion oscillates depending on the inclusion orientation and is (a) negative when the long inclusion axis is oriented in the range of 0° < *α* < 45°; and (b) positive for 45° < *α* < 90°. The presence of the rigid inclusion results in a <10% increase in the composite strength, which remains relatively constant during the whole deformation history (Figure 1h).

### 3.2 Two‐Phase Aggregate With Multiple Inclusions

#### 3.2.1 Weak Inclusions

**Figure 2:**
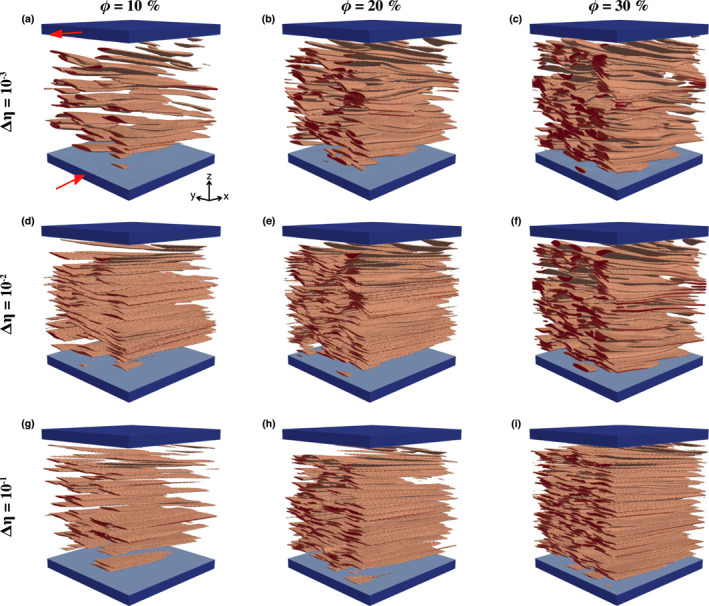
(a–i) Inclusion configuration at *γ* = 6 of shear deformation for aggregates with a weak inclusion phase. Red arrows in panel a indicate the sense of shear. See text for description of the model setup.

**Figure 3:**
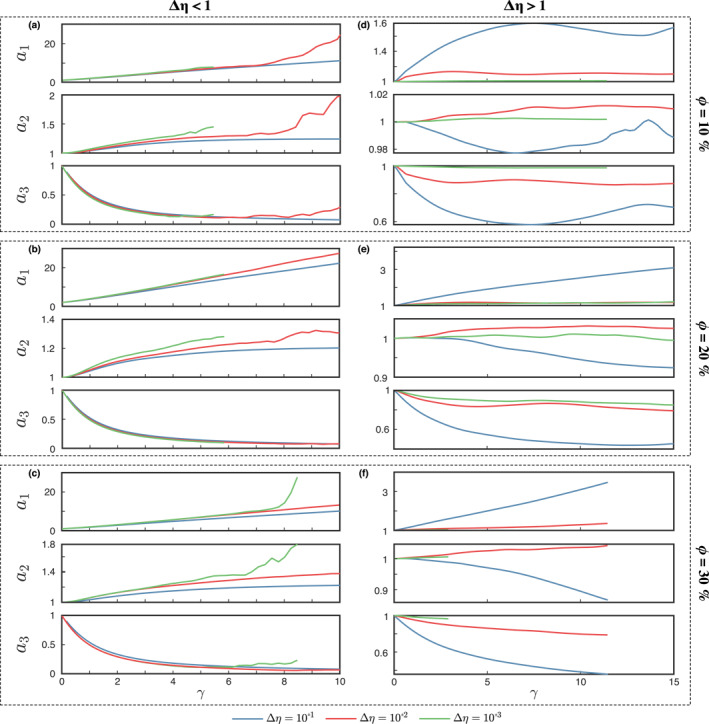
Evolution of the first *a*_1_, second *a*_2_ and third *a*_3_ principal axes of the average inclusion finite strain ellipsoid (FSEE) for (a–c) weak and (d–f) strong inclusion phase. The apparent volume increase of the inclusion phase for for viscosity constrasts larger than 2 orders of magnitude (red and green lines) observed in panels (a–c) are caused by segmentation of some inclusions.

Similar to the case of a composite with a single weak inclusion, both the *a*_1_‐axis and *a*_2_‐axis of the FSEE increase with increasing *γ* (with an almost linear trend for the former up to *γ* = 7 − 8), while the *a*_3_‐axis shows an initial rapid decrease with *γ* (Figures 3a and 3c). Given the much larger increase rate of the *a*_1_‐axis and *a*_2_‐axis, deformation results in the development of a strip‐like shape of the individual particles aligned to form an L‐S type fabric (Figures 2 and S1, and Movies S1 and S2). The *a*_2_‐axis of the FSEE increases up to ca. 20% depending on the viscosity contrast (Figure 3a–3c), reaching a near‐steady state length after ca. 600% of shear strain. The growth of the *a*_2_‐axis indicates inclusion flattening.

The shape of individual inclusions is a function of the viscosity contrast and volume fraction between the two phases. The former determines how much the weak phase can deform within the strong matrix; the latter determines the amount of inter‐crystal deformation. At low viscosity contrasts and low volume fractions of the weak phase (Figure 2g), the matrix opposes little resistance to deformation, and the inclusions, quite spaced apart, are relatively free to grow laterally without getting in contact. In this case, the inclusions are well defined ellipsoids (see Flinn diagram in Figure S3) that match with the associated FSEE.

At low viscosity contrast and high volume fractions (Figure 2i and Movie S1), the weak phase evolves into a dense network of thin, weak surfaces. The close spacing of inclusions promotes interactions, which results in gently convex/concave pseudo‐ellipsoidal inclusion shapes at high *γ*. An increase in the matrix rigidity affects the deformation of the weak phase and results in an irregular pattern of intra‐crystal deformation. This effect intensifies with volume fraction and yields: (a) a less well‐defined network of weak thin layers due to reduced flattening and lateral crystal growth; (b) increase of the inclusion curvature; and (c) local inclusion rotation around the *a*_1_‐axis (Figures 2a–2c and Movie S2). To summarize, the models show that relatively low viscosity contrasts are needed to develop very well‐defined networks of weak thin layers, while the foliated (S‐type) fabric is less well‐defined at large viscosity contrasts.

**Figure 4:**
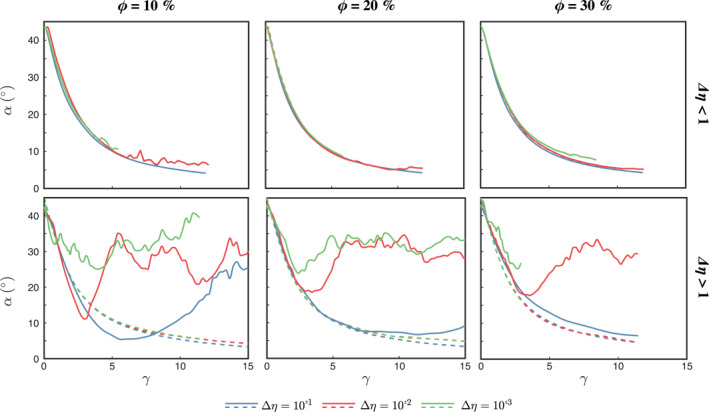
Average inclination *α* of the inclusions (solid lines) and inclination of the bulk FSE *a*_1_‐axis (dashed lines) with respect to the horizontal shear plane at different viscosity contrasts. The bulk FSE *a*_1_‐axis is not visible in the top row panel as it overlaps with the solid blue line. The bulk FSE is computed by averaging the FSE of all the Lagrangian particles, excluding the rigid plates.

The evolution of the average inclusion inclination (*α*) is shown in Figure 4, top row. At the onset of deformation, the inclusions are inclined between 40° and 45°, subparallel to the third principal stress. With increasing *γ*, the angle *α* decreases exponentially to reach a near steady state value of 5° at *γ* > 10. The observed alignment of the inclusions with the flow direction is consistent with natural observations, 2D numerical models (e.g., Dabrowski et al., 2012; Thielmann et al., 2020), and micromechanic approaches with linear and non‐linear rheology (e.g., Jiang, 2012, 2014; Yang et al., 2019). As previously stated, some inclusions experience rotation along the *X*‐axis at large viscosity contrasts; however, rotations along *Y*‐axis largely dominate. Our results further show that *α* is effectively independent of the viscosity contrast and matches the inclination of the FSEE *a*_1_‐axis of the bulk composite.

Due to severe flattening and lateral growth in models with *ϕ* = 10% and Δ*η* ∈ [10^−2^, 10^−3^], during the calculation of the FSE the distance between Lagrangian particles of the inclusions becomes large enough so that some matrix particles fill empty grid cells. This results in the artificial segmentation of the inclusion, and causes the oscillations present in some of the panels in Figures 3 and 4.

**Figure 5:**
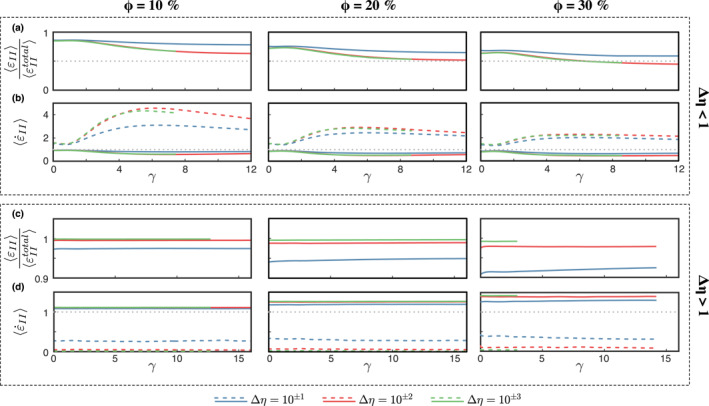
Partitioning of the volume averaged (a and c) normalized strain and (b and d) strain rate between the co‐existing material phases. The colored solid lines correspond to the matrix, colored dashed lines correspond to the inclusions, gray dashed lines in (b and d) denote the bulk strain rate that in all experiments is equal to 1.

The resulting flattening, elongation, and alignment of the inclusions with the flow direction increases the amount of weak surface in the shear plane. This has positive feedback with the relative amount of strain accommodated by the weak phase and strain progressively localizes in the weak phase as the S‐type fabric matures (Figure 5). The strain rate accommodated by the ”flat” S‐type fabric is always larger than the bulk, and the fraction of strain accommodated by the inclusions rapidly increases before stabilizing after a few per cent deformation. For a given volume fraction, the final strain partitioning depends on the viscosity contrast, with larger viscosity contrasts yielding larger amounts of strain being accommodated by the weak inclusion phase.

For *ϕ* > 10% the amount of deformation accommodated by the inclusion phase is equal or larger than the strain absorbed by the matrix. This phenomenon is not observed for Δ*η* = 10^−1^, although we cannot discard that it may occur at higher volume fractions than those considered here.

#### 3.2.2 Strong Inclusions

**Figure 6:**
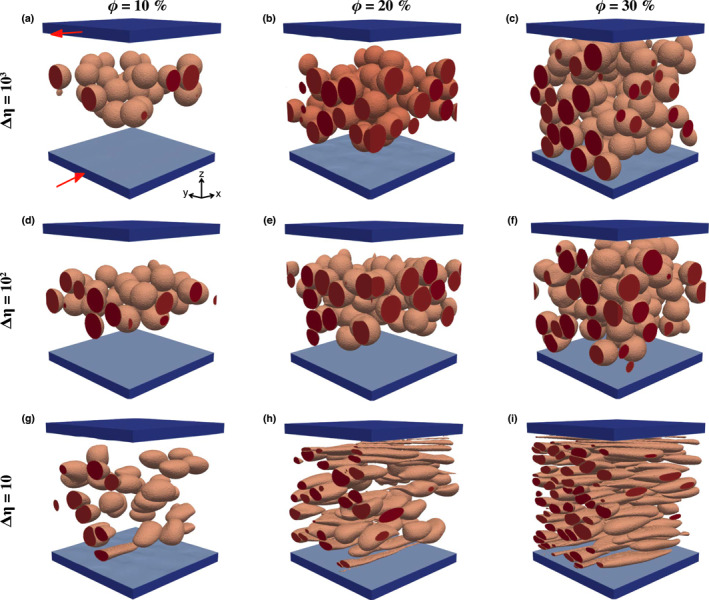
(a–i) Inclusion configuration at *γ* = 16.4 of shear deformation for composites with a rigid inclusion phase. Due to lack of convergence, the shear deformation on panel i is *γ* = 6. The red arrows in panel a indicate the sense of shear.

Strain is mostly accommodated by the weak matrix and two types of fabrics are observed depending on the viscosity contrast. The first fabric type occurs at relatively low viscosity contrast (Δ*η* < 10^2^), where the inclusions *a*_1_‐axis grows in the direction of the flow, while the *a*_3_‐axis shrinks considerably (Figures 3d–3f). The *a*_2_‐axis decreases up to 10% at high packing numbers, indicating constructional deformation. The inclusions thus deform into a prolate (cigar‐like) shape and gradually rotate toward the shear plane (Figures 4k and 4l and Movie S3). The reduced inter‐crystal spacing at *ϕ* > 10% enhances the development of the L‐type fabric (Figures 6h, 6i, S2h and S2i), because strain localizes at the contact between neighbor inclusions, enhancing the inclusions stretching. At lower *ϕ*, the L‐type fabric is less pronounced and depends on the initial spatial distribution of the inclusions, since isolated inclusions barely deform in any direction and mainly rotate along the *Y*‐axis.

The second fabric type is observed for Δ*η* ≥ 10^2^, where relative large viscosity of the inclusions inhibits large intra‐crystal deformation and the inclusions constantly rotate around the *Y*‐axis (Figure 4, bottom row), and small deviations from the initial shape are the result of interactions between inclusions. For *ϕ* < 30%, strain localizes just at the boundary with the rigid plates and the rigid inclusions concentrate toward the domain center. This phenomenon can be explained with the tendency of the system to maintain a balance in the grain dispersive pressure, which is caused by the interactions between rigid particles (Bagnold effect, Komar, 1972). The dispersive pressure increases with both the particle concentration and the velocity gradients. Thus, near the rigid boundaries the high velocity gradients must be compensated by a low rigid particle concentration, and vice versa toward the center of the composite. Clustering of the inclusion phase in a tight band is not observed at *ϕ* = 30%, where high strain localizes at the contact of the clogged inclusions (Figures S2c and S2f). For all the different combinations of Δ*η* and *ϕ*, strain is mainly accommodated by the weak matrix and remains more or less constant with increasing shear, absorbing more than 90% of the total deformation (Figures 5c and 5d).

### 3.3 Viscosity Evolution

Two‐phase aggregates are mechanically heterogeneous materials where the stiffness tensor of the composite is no longer isotropic. It is not possible to infer all the components of the anisotropic viscous tensor of the composite directly from the 3D models, but only an effective viscosity (e.g., Jiang & Bhandari, 2018):

(10) $$\eta_{eff}=\frac{\langle \tau_{II}\rangle }{2\langle {\dot{\varepsilon }}_{II}\rangle }$$

where 〈⋅〉 indicates the volume average of a given field, and the subscript *II* indicates the square root of the second invariant of an arbitrary second‐order tensor **C**, defined as $C_{II}=\sqrt{\frac{1}{2}C:C}$. In simple shear, the effective viscosity is equivalent to the shear viscosity component in the plane parallel to the shear direction, i.e., *η*_*eff*_ ≡ *η*_*xzxz*_, or *η*_*eff*_ ≡ *η*_*xz*_ in the reduced Voigt notation. To retrieve the remaining shear viscosities *η*_*xyxy*_ and *η*_*yzyz*_, the model is rotated along the *X*‐ and *Z*‐axes, respectively, at different stages of deformation to solve the instantaneous flow and compute the effective viscosity. The same approach was used by Hansen et al. (2012) to measure the normal viscosity under uniaxial extension on olivine aggregates after a certain amount of simple shear deformation. The normal viscosity components *η*_*xxxx*_, *η*_*yyyy*_ and *η*_*zzzz*_ are estimated here by imposing pure shear boundary conditions, with ***v*** = (1, 0, 0) and ***v*** = (0, 0, −1) prescribed at the boundary on the plane *x* = 1 and at the top of the domain, respectively, and free‐slip boundary condition at the remaining boundaries of the domain. In this latter case, the rigid plates located at the top and bottom of the model are removed. The inclusions do not align perfectly with the horizontal plane and, therefore, the anisotropic viscous tensor contains non‐zero values in the off‐diagonal blocks, as well as in the off‐diagonal indices of the lower diagonal block. However, direct retrieval of these components of the viscous tensor from the numerical models is not possible.

The numerical convergence of the rotated models is difficult to achieve as the result of large viscosity discontinuities, pre‐existing complex morphology, and lack of a good estimate of the flow solution. Furthermore, some inclusions may split in two across the bottom and top non‐periodic boundaries after rotation. In particular, pure shear models fail to converge in models with viscosity jumps larger than one order of magnitude, and even in some cases with low viscosity contrast. Only converged models are shown in this section. In Appendix A we demonstrate that the normal and shear components of the anisotropic tensor can be recovered from 3D models with a simple morphology where a fully converged flow solution is achieved.

#### 3.3.1 Weak Inclusions

**Figure 7:**
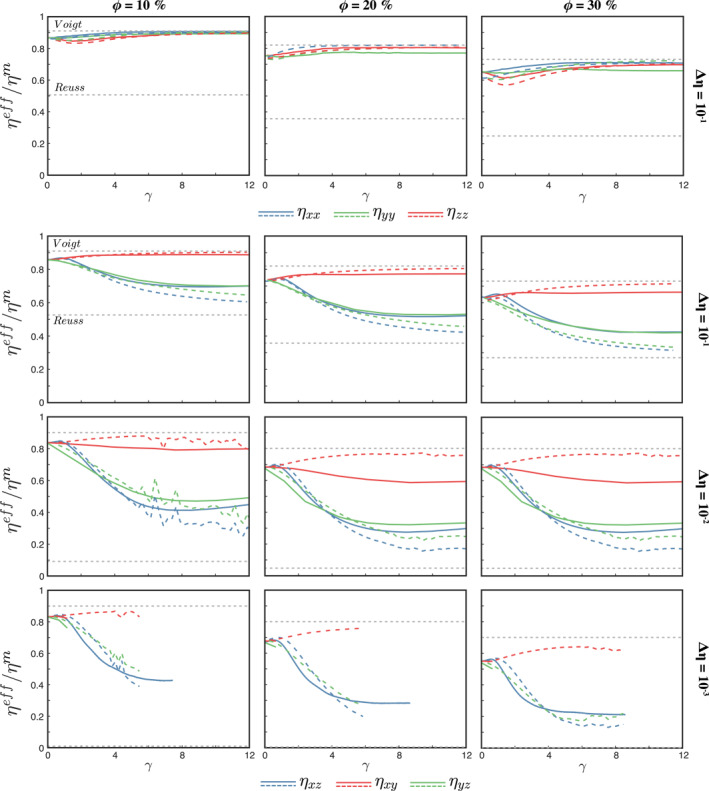
Evolution of the anisotropic viscous tensor main components for two‐phase aggregate with a weak inclusion phase. Solid lines correspond to the viscosity evolution from the 3D models; colored dashed lines correspond to the viscosity computed by the differential effective medium (DEM), using the average fabric shape and orientation of the 3D models. Gray dashed lines are the Reuss and Voigt lower and upper bounds, respectively. The normal components of the viscous tensor (top panels) are shown only for converged flow solutions.

The shear‐parallel viscosity exhibits the same trend in all simulations (Figure 7), with a short initial stage of hardening followed by rapid weakening and finally a new stage of gentle hardening at large deformation. The first hardening occurs at 0.5 < *γ* < 1 and is related to the transition of the initial spherical shape of the weak inclusions to *σ*_3_‐parallel ellipsoids. This produces a disturbance in the otherwise quasi shear‐parallel flow and results in a 3%–5% increase of the bulk viscosity. This initial hardening has been documented in 2D forward models with inclusion with spherical (Dabrowski et al., 2012) and random (Thielmann et al., 2020) shapes, in self‐consistent micro‐mechanical models with varying‐shape ellipsoids and power‐law rheology and flow fields more complex than simple shear (Jiang, 2014).

After the viscosity peak is reached, the models experience a phase of intense weakening, where the weakening rate is controlled principally by, and increases with, viscosity contrast. For example, models with Δ*η* = 10^−3^ and 10^−1^ require about 500% and 1,600% of shear deformation, respectively, to reach the maximum weakening. After this point, the composite does not reach a steady‐state of constant viscosity, but a stage of slight hardening. We infer that this last hardening stage is a combination of three mechanisms. First, even though our models do not exhibit a general loss of resolution, the edges of the inclusions may become thinner than the vertical spacing of the grid at large strain and introduce some numerical artifacts (Thielmann et al., 2020). Second, given an ellipsoidal inclusion with angle *β* < 45° between their second principal semi‐axes and the horizontal plane, any rotation so that *β* ≤ *β* ^new^ ≤ 45° yields a stronger aggregate. At large strain, the inclusions develop different degrees of non‐homogeneous internal rotation with the vorticity axes given by the shear‐parallel direction (*X*‐axis) that creates planes within the inclusion at a higher inclination with respect to the horizontal plane (Figure 2), thus arguably hardening the aggregate. And third, segmentation of severely sheared inclusions at large strain. Quantifying the contribution of these mechanisms to the total hardening would require running the models with considerably denser particle density and finer grid. In the latter case, adaptive meshes should be used, as the problem soon becomes computationally prohibitive with regular grids. On the other hand, normal stress is mainly supported by the matrix and, therefore, the normal viscosity components harden as the inclusions flatten, tending toward the Voigt upper bound (Figure 7).

The dashed lines in Figure 7 represent the shear‐parallel viscosity calculated from the DEM Equation 5 by employing the average inclusion shape (FSEE) and subsequent correction of the average inclination of the fabric. The DEM matches well both initial and peak shear viscosity components, as well as the weakening stage of *η*_*xz*_ and *η*_*yz*_, but overestimates the maximum amount of weakening. The *η*_*xy*_ component is also well predicted by the DEM at low viscosity contrast (Δ*η* = 10^−1^). At larger viscosity contrasts, the DEM fails to predict the observed softening behavior and predicts a phase of hardening for *η*_*xy*_. We hypothesize that the observed weakening is caused by lateral distortions of the ellipsoidal shape (Figures 2b, 2c, 2e, and 2f) that are not captured by the DEM. As in Figures 3 and 4, the wiggles in the DEM curves in the middle and bottom panels of the left‐hand‐side column in Figure 7 are caused by poorly resolved FSE related to the segmentation of weak inclusions.

**Figure 8:**
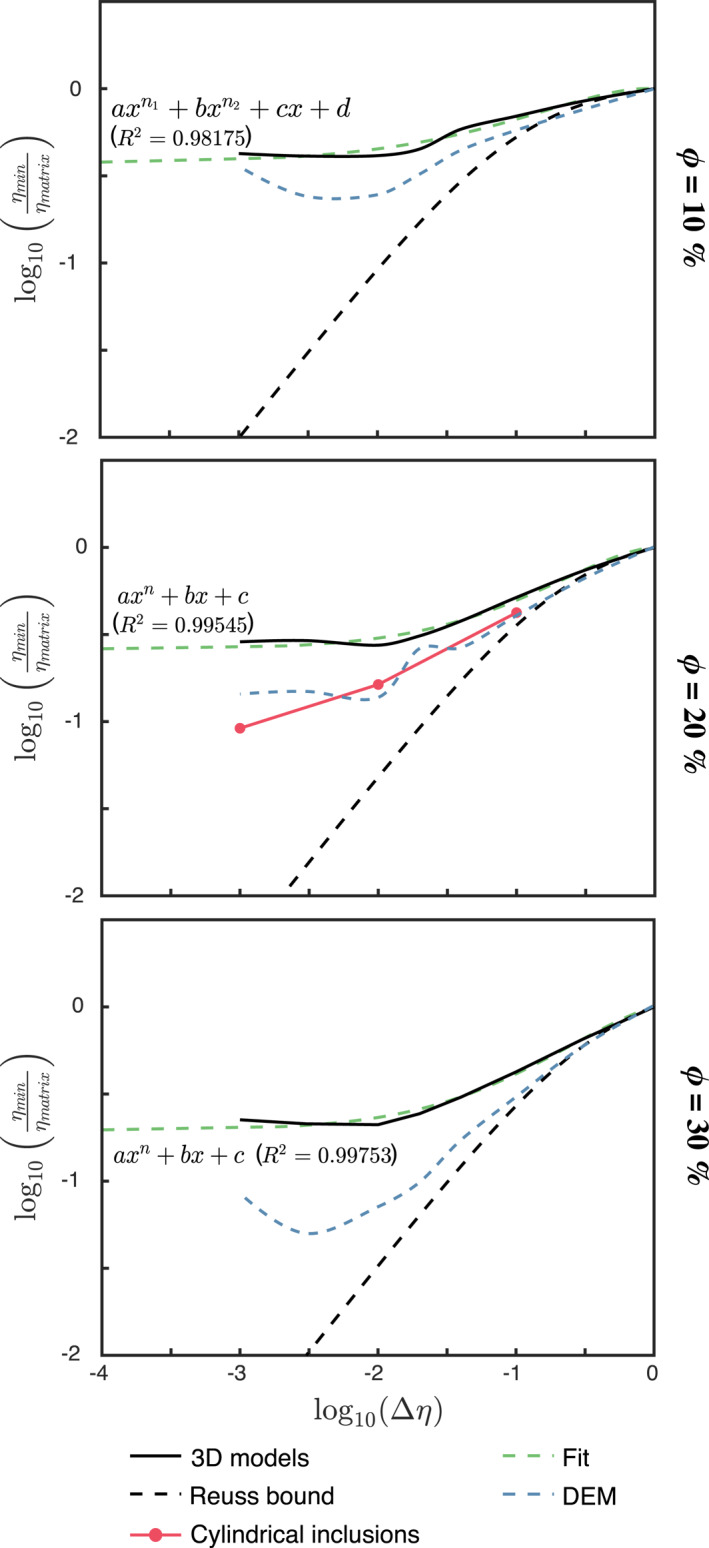
The minimum composite viscosity normalized with respect to the matrix viscosity (equivalent to 1 − *ω*) as a function of the viscosity contrast. The black solid line correspond to the 3D models, the dashed black line is the Reuss bound, the dashed blue lines are the predictions from the differential effective medium (DEM), the dashed green line is the fit to the 3D models, and the solid red line with red circles is for 3D models with cylindrical inclusions.

The maximum weakening of the composite relative to the matrix viscosity is defined as *ω* = 1 − min(*η*_*xz*_)/*η* ^*m*^ (0 < *ω* < 1). Figure 8 shows in logarithmic scale 1 − *ω* = min(*η*_*xz*_)/*η* ^*m*^ at different combinations of Δ*η* and *ϕ*. For any given *ϕ*, *ω* exponentially increases with increasing matrix viscosity until a maximum at Δ*η* ≈ 10^−2^ where *ω* ≈ 60–80%. A small reduction of *ω* is observed for larger Δ*η*. The irregular patterns of inclusion deformation (e.g., inclusion tilting and convex/concave inclusion shapes: Figures 2c, 2f, and 2i) are responsible for the reduced weakening. Models with $\Delta \eta \in \left[1/3,1/25,1/50,1/330\right]$ were run to produce higher‐resolution curves of the maximum weakening.

It is important to note that *ω* differs from the structural weakening *ω*_struct_, where the latter refers to weakening of the bulk aggregate relative to the initial, undeformed state (i.e., $\omega_{struct}=1-\min\left(\eta_{xz}\right)/\eta_{xz}^{0}$). Structural weakening (*ω*_struct_), always lower than the weakening normalized against the matrix viscosity (*ω*), oscillates between 30% (for Δ*η* = 10) and up to 60% (for larger Δ*η*).

The dashed blue line in Figure 8 represents the reduction in the bulk shear viscosity obtained by solving the DEM Equation 5. As previously discussed in Section 3.3, this semi‐analytical equation predicts a stronger weakening of the composite in comparison to our 3D models. The misfit between the weakening from the DEM and the numerical models increases with Δ*η* and *ϕ*, since the characterization of the average fabric becomes more difficult.

#### 3.3.2 Strong Inclusions

**Figure 9:**
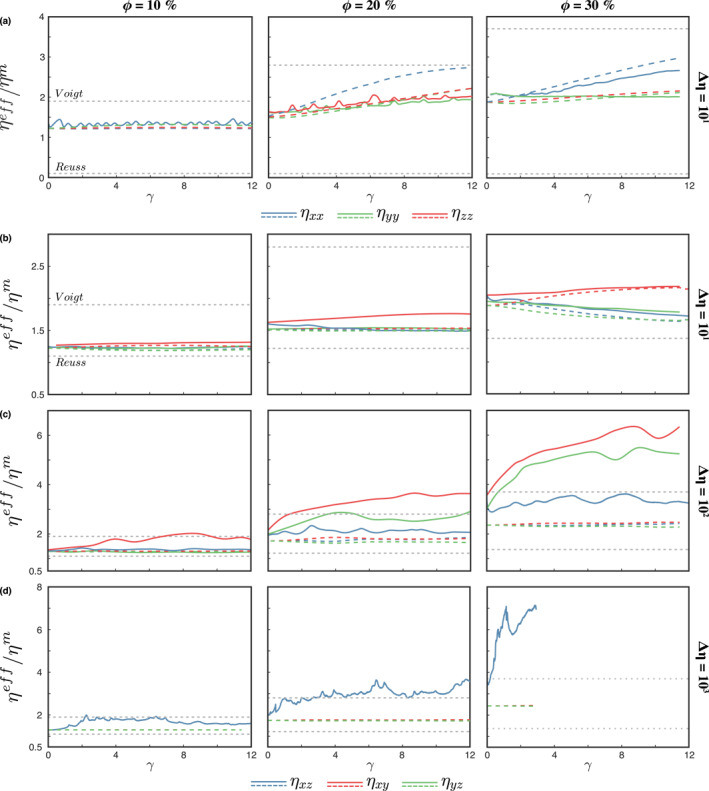
Evolution of the anisotropic viscosity tensor of a two‐phase aggregate with a strong inclusion phase. Solid lines represent the viscosity retrieved from forward numerical simulations. Dashed lines are the *η*_*xz*_ obtained from the differential effective medium (DEM) using the average shape of the weak phase as input and corrected for the average inclusion inclination. Gray dashed lines are the Reuss and Voigt lower and upper bounds, respectively. The normal components of the viscous tensor (top panels) are shown only for converged models.

The presence of rigid inclusions only moderately affects the effective strength of composites. A viscosity contrast of 3 orders of magnitude results in an increase of no more than 5–6 times the viscosity of the matrix, while the impact of the rigid inclusions is considerably less pronounced at lower Δ*η*. Shear viscosity of composites with L‐type fabrics (Figure 9, second row) exhibit slight strain softening, related to the rotation of the cigar‐shaped inclusions from a *σ*_3_‐parallel orientation to a stable position at few degrees off the flow direction. The resulting fabric is well characterized by the FSEE and, consequently, the DEM predicts well the shear‐parallel viscosity with great accuracy. The L‐type fabric no longer develops at Δ*η* > 10, where particle rotations and small inclusion interactions are reflected in the oscillatory evolution of the effective viscosity (Figure 9, third and fourth rows). This is consistent with previous studies based on a multi‐scale self‐consistent micro‐mechanical approach with power‐law rheology (Yang et al., 2019), with the difference that the upper bound of viscosity contrast at which L‐type fabrics develop was set at Δ*η* = 5. These models show a clustering of the inclusions in a tight band at the center of the model domain that reduces the space between inclusions, resulting in a jammed aggregate where high stress localizes inside the inclusions and yields a viscosity that exceeds the Voigt upper bound. The DEM does not accurately predict the strength of jammed configurations and significantly underestimates the anisotropic viscosity.

## 4 Up‐Scaling to Large Scales: Fabric Parameterization

The scale of the fabrics developing in multi‐phase composites, aimed at simulating rock fabrics, is several orders of magnitude smaller than geological structures at the regional‐to‐plate tectonics scale. Therefore, it is computationally not feasible to include realistic multi‐phase materials in numerical codes given the currently available computational power. As a consequence, mechanical anisotropy linked to the development of SPO cannot be incorporated in numerical models and materials are typically approximated as isotropic bodies.

Given the good match between the extrinsic viscous tensor obtained from forward numerical simulations and the DEM, the latter could be used to approximate the SPO‐related viscous anisotropy of two‐phase aggregates with a given fabric shape and orientation. In this section we propose a parameterization for the simulated 3D fabric as a function of parameters that might be either known or easily computed by geodynamic codes. Once the average composite morphology and orientation are estimated, the viscous tensor can be approximated by first solving the DEM Equation 5, and then rotating the anisotropic tensor with the angle *α* around the *Y*‐axis.

### 4.1 Average Fabric Shape

#### 4.1.1 Weak Inclusions

In the simplest case where the matrix and the inclusions have equal viscosity, the latter are perfectly aligned and their shape is given by the bulk FSE, which in simple shear and plane strain implies that $a_{1}/a_{2}=a_{2}/a_{3}=\sqrt{\exp\left(2\sinh^{-1}\left(\gamma /2\right)\right)}$. This expression is linear in the Flinn diagram and plots over the diagonal. For aggregates with weak inclusions we observe that the path of the FSEE is also quasi‐linear in the Flinn space, and is approximately linearly proportional to the diagonal (i.e., bulk deformation), at least for the strain range in our models. Thus the fabric can be approximated as:

(11) $$r_{i}=\zeta_{i}A$$

where *r*_*i*_ = log _10_(*a*_*i*_/*a*_*i*+1_) and $A=\log_{10}\left(a_{i}^{bulk}/a_{i+1}^{bulk}\right)$, with *i* = 1, 2, are the respective ratios of the FSEE and bulk FSE semi‐axis, and *ζ*_*i*_ is the proportionality constant. The evolution of the ratio between the intermediate and shortest semi‐axis of both FSEE and bulk FSE is approximately the same (i.e., *r*_2_ ≈ *A*) and only the parameterization of *r*_1_ is necessary. The fitting coefficient *ζ*_*i*_ = *ζ*(*ϕ*, Δ*η*) slightly depends on the morphology and rheology of the aggregate (Table 1) and decreases as the inclusion volume fraction and viscosity contrast increase, reflecting that inclusion flattening is enhanced by inclusion interactions in composites with strongly varying mechanical properties.

**Table 1:** Fitting Parameters *ζ*, *ξ*, *χ*, *θ*, *ψ*, *λ*, and Their Associated R^2^ Values for the Fits Corresponding to Average Shape (*r*_1_, Equation 11), Average Fabric Inclination (*α*, Equation 14), and Maximum Weakening of the Shear Viscosity Component (*ω*, Equation 12)

	*ζ*	*ξ*	*χ*	*θ*	*ψ*	*λ*	*R* ^2^
*r*_1_(*ϕ* = 10%, Δ*η* = 10^−1^)	0.843781	‐	‐	‐	‐	‐	0.985
*r*_1_(*ϕ* = 20%, Δ*η* = 10^−1^)	0.793576	‐	‐	‐	‐	‐	0.996
*r*_1_(*ϕ* = 30%, Δ*η* = 10^−1^)	0.752338	‐	‐	‐	‐	‐	0.994
*r*_1_(*ϕ* = 10%, Δ*η* = 10^−2^)	0.715399	‐	‐	‐	‐	‐	0.994
*r*_1_(*ϕ* = 20%, Δ*η* = 10^−2^)	0.774659	‐	‐	‐	‐	‐	0.999
*r*_1_(*ϕ* = 30%, Δ*η* = 10^−2^)	0.704054	‐	‐	‐	‐	‐	0.992
*r*_1_(*ϕ* = 10%, Δ*η* = 10^−3^)	0.687903	‐	‐	‐	‐	‐	0.988
*r*_1_(*ϕ* = 20%, Δ*η* = 10^−3^)	0.732392	‐	‐	‐	‐	‐	0.998
*r*_1_(*ϕ* = 30%, Δ*η* = 10^−3^)	0.709184	‐	‐	‐	‐	‐	0.989
*α*(*ϕ* = 10%, Δ*η* = 10^−1^)	0.921804	‐	‐	‐	‐	‐	0.998
*α*(*ϕ* = 20%, Δ*η* = 10^−1^)	1.007913	‐	‐	‐	‐	‐	0.988
*α*(*ϕ* = 30%, Δ*η* = 10^−1^)	0.983557	‐	‐	‐	‐	‐	0.998
*α*(*ϕ* = 10%, Δ*η* = 10^−2^)	1.002619	‐	‐	‐	‐	‐	0.986
*α*(*ϕ* = 20%, Δ*η* = 10^−2^)	0.945960	‐	‐	‐	‐	‐	0.981
*α*(*ϕ* = 30%, Δ*η* = 10^−2^)	1.079322	‐	‐	‐	‐	‐	0.984
*α*(*ϕ* = 10%, Δ*η* = 10^−3^)	1.000936	‐	‐	‐	‐	‐	0.975
*α*(*ϕ* = 20%, Δ*η* = 10^−3^)	0.933471	‐	‐	‐	‐	‐	0.974
*α*(*ϕ* = 30%, Δ*η* = 10^−3^)	1.095165	‐	‐	‐	‐	‐	0.957
*ω*(*ϕ* = 10%)	0.903221	1.20365	−1.148182	0.3735	0.698558	0.6619	0.981
*ω*(*ϕ* = 20%)	43.646559	−42.907958	0	0.261095	0.984129	1	0.995
*ω*(*ϕ* = 30%)	40.628095	−39.823767	0	0.196188	0.985677	1	0.997

*Note*. The fitting parameters found in this table correspond to composites with weak inclusions.

Combining Equation 11 with the DEM to estimate the anisotropic viscosity has to be done with caution, as the latter yields a considerably weaker aggregate at large strain compared to the forward models (Figures 7 and 8). As a work‐around, the maximum weakening observed in the 3D models can parameterized and used as a lower cut‐off for the viscosity predicted by the DEM. The maximum weakening shows a non‐linear relationship with the bulk deformation and physical parameters of the aggregate, which we find to be well‐estimated (*R* ^2^ > 0.98) as:

(12) $$\omega \left(\phi \right)=1-\zeta \left(\phi \right)\Delta \eta^{\psi \left(\phi \right)}+\xi \left(\phi \right)\Delta \eta^{\lambda \left(\phi \right)}+\chi \left(\phi \right)\Delta \eta +\theta \left(\phi \right)$$

where the fitting coefficients *ζ*, *ξ*, *χ*, *θ*, *ψ*, *λ* depend on the volume fraction of the weak phase and are shown in Table 1. Extrapolations to larger Δ*η* must be taken with caution, as the hardening behavior of *ω* is not well‐reproduced by Equation 12 and additional data is needed to further understand this effect.

#### 4.1.2 Strong Inclusions

**Figure 10:**
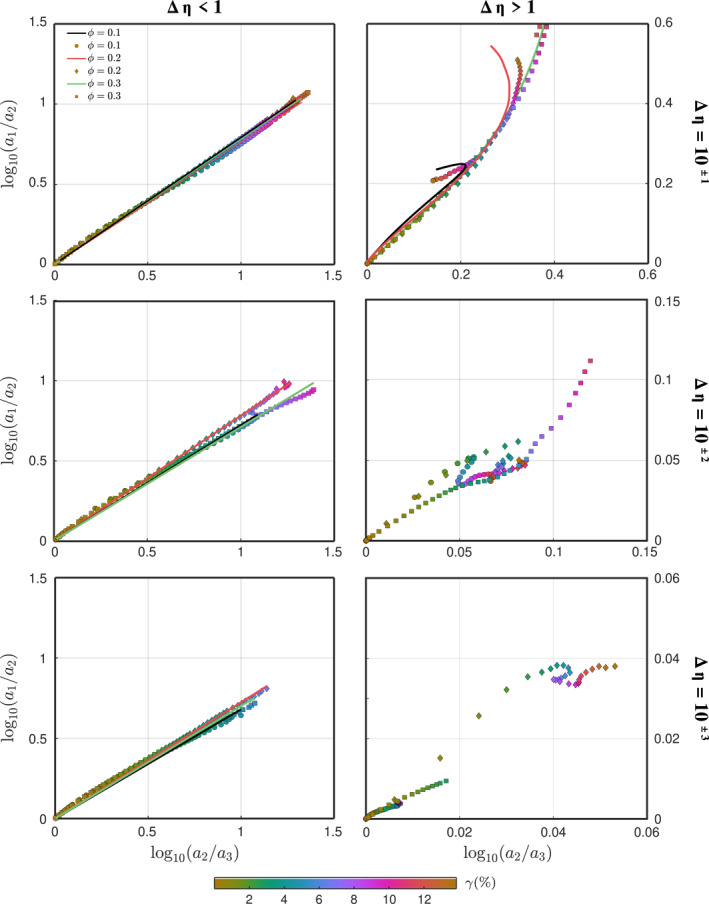
Flinn diagrams. Scattered symbols describe the average inclusion shape of the 3D models at different *γ*. The solid lines are the average inclusion shape predicted by Equations 11 and 13.

The deformation path of the FSEE of hard inclusions is complex and it is no longer proportional to bulk deformation (Flinn diagram diagonal). Additionally, *r*_2_ ≉ *A* and two parameterizations are needed to fully estimate the fabric. After testing several expressions, we find the following third order polynomial to reproduce well (*R* ^2^ > 0.97; Figure 10) the fabric evolution:

(13) $$r_{i}\left(\phi ,\Delta \eta \right)=\zeta_{i}\left(\phi ,\Delta \eta \right)+\xi_{i}\left(\phi ,\Delta \eta \right)A+\chi_{i}\left(\phi ,\Delta \eta \right)A^{2}+\theta_{i}\left(\phi ,\Delta \eta \right)A^{3}$$

where the fitting coefficients *ζ*, *ξ*, *χ*, *θ* (Table 2) are found via linear regression. We found difficult to produce an accurate parametrization of the fabric for Δ*η* > 10, as small inter‐crystal interactions yield a highly non‐linear deformation path. Nonetheless, these inclusions barely deform and maintain a near‐spherical shape at large strain (Figure 6). Therefore, they can be safely considered as such.

**Table 2:** Fitting Parameters *ζ*, *ξ*, *χ*, *θ* and Their Associated R^2^ Values, for the Fits Corresponding to the Average Shape (*r*_*i*_, Equation 11) and Inclusion Orientation (*α*, Equation 14) of Aggregates With a Strong Inclusions

	*ζ*	*ξ*	*χ*	*θ*	*R* ^2^
*r*_1_(*ϕ* = 10%, Δ*η* = 10)	−0.006131	0.458978	−0.125306	−0.083918	0.957
*r*_2_(*ϕ* = 10%, Δ*η* = 10)	0.000512	−0.319324	0.185224	−0.306101	0.971
*r*_1_(*ϕ* = 20%, Δ*η* = 10)	−0.000913	0.389964	−0.056822	0.1010262	0.999
*r*_2_(*ϕ* = 20%, Δ*η* = 10)	0.002493	0.296955	0.271349	−0.2545942	0.997
*r*_1_(*ϕ* = 30%, Δ*η* = 10)	0.000295	0.372009	−0.070759	0.2408665	0.997
*r*_2_(*ϕ* = 30%, Δ*η* = 10)	0.005547	0.266632	0.271325	−0.1762204	0.997
*α*(*ϕ* = 20%, Δ*η* = 10)	1.020180	‐	‐	‐	0.951
*α*(*ϕ* = 30%, Δ*η* = 10)	1.043904	‐	‐	‐	0.973

### 4.2 Average Fabric Orientation

The components of the viscosity tensor vary with the relative orientation of the fabric with respect to the coordinate system of choice. Thus a good prediction of the fabrics must be completed with the orientation of the fabric with respect to the reference coordinate system. Our models show that fabric rotations off the *Y*‐axis are negligible, and therefore only the angle *α* between the longest axis of the FSEE and the horizontal (XY) plane is relevant.

#### 4.2.1 Weak Inclusions

The orientation of the fabric is linearly proportional to the orientation of longest axis of the bulk FSE:

(14) $$\alpha =\zeta \left(\phi ,\Delta \eta \right)\alpha^{bulk}$$

yielding *R* ^2^ > 0.95. The values of the proportionality constant (Table 1) are *ζ* ≈ 1, exemplifying that as already discussed in Section 3.2, the mismatch between the fabric orientation and the orientation of the longest axis of the bulk FSE is minimum (Figure 4), so that *α* ≈ *α* ^bulk^ is a good approximation of the inclusions orientation. Alternatively, in plane strain deformation and again assuming the alignment of the fabric with the bulk FSE, the analytical solution from McKenzie (1979) can be used:

(15) $$\alpha =\frac{1}{2}\tan^{-1}\left(\frac{\Gamma \tanh\left(\sqrt{1-\Gamma^{2}}\dot{\varepsilon }t\right)}{\sqrt{1-\Gamma^{2}}}\right)$$

where $\Gamma =\Omega /\dot{\varepsilon }$ is the vorticity number, with Ω being the magnitude of the rotational component about the *Y*‐axis.

#### 4.2.2 Strong Inclusions

Hard inclusions exhibit a constant rotational behavior where the small amount of inter‐crystal deformation introduces negligible disturbances in the evolution of the viscosity tensor. Only at *ϕ* > 10% and low Δ*η* (about one order of magnitude), rigid inclusions develop an L‐type fabric whose orientation is predicted by Equation 14 with the fitting coefficients in Table 2. Because of heterogeneous particle interactions at *ϕ* = 10% and Δ*η* = 10, some elongate, while others adopt a rotational behavior (Figures 4 and 6g). To produce an accurate estimate of the orientation for this case, the forward model should run to larger deformation to study whether the aggregate fully develops an L‐type fabric or reaches an stationary average rotational state.

## 5 Discussion

The kinematics of porphyroclasts are known to be sensitive to the rheology of the surrounding matrix (e.g., Passchier & Sokoutis, 1993). Additionally, rock‐analogue experiments of isolated ellipsoidal and rhomboidal rigid inclusions suggest that a slipping boundary between the inclusion and the matrix is essential to attain a stable configuration (Mancktelow et al., 2002), otherwise the inclusion continuously rotates. These observations are in agreement with our models, with perfect coherence between the inclusions and the matrix, for (a) an isolated rigid inclusion, and (b) rigid inclusions of at least two orders of magnitude stiffer than the matrix, where a stable configuration is not reached. At moderately low viscosity contrast (Δ*η* < 10^2^), instead, we observe the development of a strong SPO. The fabric development rate is strongly related to the volume fraction of the rigid phase: in densely populated aggregates, channels of high‐strain‐rate, weak matrix form in between nearby inclusions, inhibiting the rotation and accelerating the inclusion elongation. Large finite strain (*γ* ≫ 20) is required to reach a stable configuration at low volume fractions. The inclination of the SPO increases with increasing Δ*η* (e.g., about 8° at Δ*η* = 10, and 25° at Δ*η* = 50, both with *ϕ* = 30%). Therefore, the angle *α* may be used as a proxy for the aggregate rheology. We note that rheological non‐linearities, such as dislocation creep or clast fracturing, and the degree of coherence between matrix and inclusions (Ceriani et al., 2003; Mancktelow et al., 2002) may significantly influence the kinematic behavior of the inclusions. These parameters should be included in future numerical simulations to better constrain the dynamics of matrix‐inclusions systems.

When *ϕ* < 30%, rigid particles tend to concentrate away from the sliding rigid plates and toward the center of the model domain due to the Bagnold effect (Komar, 1972; Figures 6a, 6b, 6d, and 6e). This is consistent with the progressively larger concentration of phenocrystals and clasts observed toward the center of, respectively, magmatic dikes (Komar, 1972) and pseudotachylite veins (Di Toro & Pennacchioni, 2004). However, the latter result is not applicable to deforming viscous rocks where rigid walls are absent and a more homogeneous distribution of the harder inclusions is expected (as in models with *ϕ* = 30%; Figures 6c, 6f–6i). In contrast, weak inclusions are not affected substantially by the model boundary conditions and setup, and the modeled structures are likely representative of real composites. For instance, the L‐S fabrics obtained in models with weak inclusions and low *ϕ* (Figures 2a, 2d, and 2g) are strikingly consistent with those of bubble‐bearing magma sheared at large strains (Caricchi et al., 2011).

More generally, the modeled fabrics display several similarities with those observed in natural and experimentally deformed samples, which suggests the 3D models are capable of capturing, at least to a first order, the mechanical behavior of these composites. For example, intensively sheared gneiss rocks of the continental crust are frequently characterized by elongated ribbons of harder feldspar grains surrounded by flattened and laterally irregular domains of weaker mica and quartz. This might indicate that in these cases the viscosity contrast between these minerals is $<10^{2}$ (Figures 6h and 6i) and the extrinsic viscous anisotropy is small (Figures 9a, and 9b). The numerical models reproduce the fabrics, strain weakening and strain partitioning in sheared synthetic samples representative of two‐phase mantle aggregates (e.g., Girard et al., 2016), but a direct comparison to mantle rocks samples is difficult, as outcrops of the latter are generally part of the exhumed lithosphere, and hence they are not entirely representative of the deep and hot mantle where a more diffused and long‐lasting deformation accommodated by high‐*T* creep takes place.

Recent numerical studies demonstrated that anisotropy related to lattice preferred orientation (LPO) in olivine crystals can yield a weakening of about *ω* = 30% in the shear direction, and up to one order of magnitude viscosity variations depending on the dominant slip system (Király et al., 2020). The weakening is thus about a half of the predicted by SPO in our models, while the directional variations linked to LPO claimed by Király et al. (2020) can be up to two larger than what observed in our two‐phase aggregates and DEM models (Figure 7). This implies that an aggregate with mechanically anisotropic crystals should be more susceptible to changes in flow directions than when only isotropic SPO‐related fabrics are present.

2D numerical simulations (Dabrowski et al., 2012; Thielmann et al., 2020) show that strain progressively localizes in the weak inclusions as they elongate under simple shear deformation, considerably weakening the bulk composite before developing a network of fully interconnected weak planes. This implies a fabric maturity‐dependent transition from a load‐bearing framework to a network of interconnected weak layers. The weakening resulting from the compositional layer has been invoked to inhibit the mixing of material in the lowermost mantle (Ballmer et al., 2017) and enhance the connection between the upper and lower mantle through narrow conduits of rapidly ascending hot material (Christensen, 1987). The layering and strain‐softening behavior of composites is well‐reproduced by 3D models. However, our results suggest that weakening related to compositional layering is considerably less than reported by 2D plane‐strain simulations.

**Figure 11:**
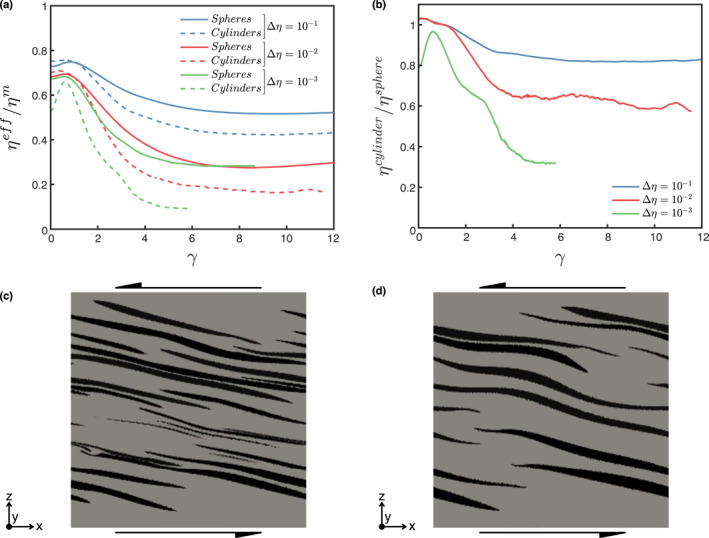
(a) Evolution of the normalized effective viscosity for two‐phase aggregates with spherical (solid lines) and cylindrical (dashed lines) inclusions. (b) Evolution of the ratio between the effective viscosity of two‐phase aggregates with cylindrical and spherical inclusions. Cross section along the plane orthogonal to the *Y*‐axis for (c) spherical and (d) cylindrical inclusions for Δ*η* = 10^−2^ at *γ* = 4.2. The gray and black colors indicate the matrix and inclusion phases, respectively, and the black arrows indicate the sense of shear. The volume fraction of the weak phase is 0.2.

Plane strain implies that the model is infinitely continuous along the direction orthogonal to the 2D cross‐section. In other words, the inclusions in 2D representations of two‐phase aggregates are continuous fibers of infinite length. The maximum reduction in normalized bulk viscosity, the evolution of the shear‐parallel viscosity and the inclusion morphology are compared in Figures 8b and 11 for an aggregate with initially spherical inclusions and an aggregate with full‐width cylindrical inclusions (*ϕ* = 20% and different Δ*η*). Both model set‐ups yield comparable cross‐section morphologies (Figures 11c and 11d), which are comparable to the 2D morphologies in Dabrowski et al. (2012) and Thielmann et al. (2020).

The weakening observed in the model with cylindrical inclusions (Figure 11a) is also within the range of weakening of 2D models, and amounts to max. 60–80%. However, perfect inter‐connectivity in the *Y*‐direction has a strong influence on the effective viscosity of the composite and the strength of models with a more realistic 3D set‐up quickly diverges from the plane‐strain approximation at *γ* > 2. Plane strain models overestimate the amount of weakening by about 25% at Δ*η* of one order of magnitude. The overestimation of weakening increases exponentially with increasing Δ*η* between coexisting phases, predicting a three‐times weaker composite for Δ*η* of three orders of magnitude. Structural weakening *ω*_struct_ (i.e., ratio between the bulk minimum and initial viscosities) is also significantly exaggerated by plane strain, which yields between 60% and 85% of *ω*_struct_ at Δ*η* = 10^−1^ and Δ*η* = 10^−3^, respectively. In contrast, only about 30% and 60% of *ω*_struct_ is predicted by models with spherical inclusions. This comparison shows that while 2D models are a valid tool to provide first‐order insights on highly 3D problems, the quantitative results should be taken with caution and further 3D studies with increasing degrees of complexity are required to better constrain the dynamics of multi‐phase aggregates.

**Figure 12:**
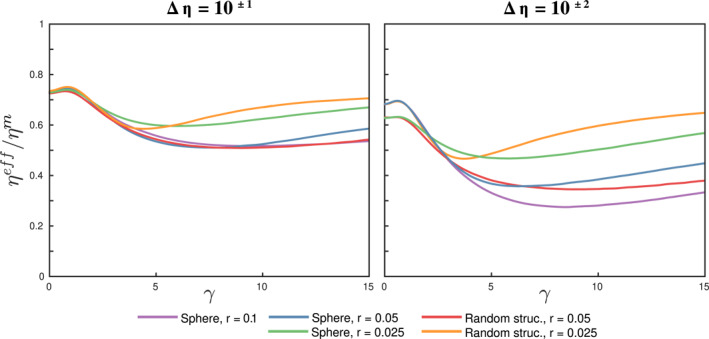
Evolution of the normalized effective viscosity with increasing deformation for aggregates with spherical inclusions with equal radius 0.1, 0.05, and 0.025, and heterogeneous random structures with average radius 0.05, and 0.025. The volume fraction of the inclusion phase is 20%.

In this work, we considered only two‐phase aggregates with isolated spherical inclusions of equal dimensionless radius 0.1. As discussed in Section 3.3, although this geometry is a good proxy for many relevant cases, two‐phase aggregates may be comprised by overlapping inclusions forming heterogeneous clusters of random shape, such as the synthesized ferropericlase samples from Yamazaki et al. (2009). To assess the effect of overlapping inclusions and inclusion size, we ran two additional sets of experiments with: (a) spherical inclusions of equal radius 0.05 and 0.025; and, (b) random heterogeneous random media with a morphology such that the aggregate is statistically isotropic (Figure S5). To generate the latter models, we use the statistical approach developed by Thielmann et al. (2020), and the average inclusion radii is 0.05 and 0.025. These models are run only with Δ*η* = 10^−1^ and 10^−2^, and *ϕ* = 20%. The evolution of the normalized effective viscosity (Figure 12) shows that the size and shape distribution of the inclusion phase does not dominate the aggregate rheology, which is mainly determined by the volume fraction of the weak phase. The early onset of post‐weakening hardening in aggregates with inclusions of *r* = 0.025 is triggered by inclusion segmentation and/or loss of vertical resolution (Figure S5d).

The extrinsic anisotropy of two‐phase composites with weak inclusions, and strong inclusions with low viscosity variations between coexisting phases, is well‐predicted by the DEM. The estimated viscous tensor given by the DEM is particularly accurate at low strain. The DEM overestimates the amount of weakening by a factor of 0.1–0.15 at large strain because deformation is not equally partitioned among individual inclusions and the average fabric defined by the FSEE does not perfectly represent the morphology of the composite. The fabrics developed in our models are well predicted as a function of bulk deformation. This, combined with the DEM, provides a simple framework to forecast the extrinsic anisotropy for a wide range of geological applications. The set of parameterizations proposed here are calibrated for a linear Newtonian rheology, simple shear deformation, and relatively low finite deformation. The validity of the proposed parameterization under different conditions (e.g., for composites deforming by power‐law creep such as dislocation creep, or deforming during different bulk strain geometry) is to be explored. On top of this, the deformation fabric may be destroyed, causing an increase in the bulk viscosity, by different microstructural processes: (a) post kinematic annealing at low strain; (b) phase mixing related to dissolution/precipitation, nucleation and cavitation processes (e.g., Skemer et al., 2010; Kilian et al., 2011) at high strain; and, (c) as a result of partial melting of the aggregate.

## 6 Conclusions

We present a method to predict the viscous anisotropy of two‐phase aggregates in simple shear deformation by combining numerical simulations and analytical solutions. Numerical models are used to simulate the development of 3D fabrics and quantify viscous anisotropy linked to SPO of two‐phase (matrix and inclusion) aggregates. A range of geologically relevant inclusion volume fractions and viscosity contrasts have been considered.

Weak inclusions quickly flatten with increasing bulk strain and grow laterally forming a complex network of weak thin layers where most strain localizes. The aggregate is progressively weakened in the flow direction, reducing the strength of the aggregates by up to 80% relative to that of the matrix at moderate‐to‐high volume fractions of the weak inclusions and large viscosity contrasts. The structural weakening of the aggregate is lower, reaching a maximum value of about 60% with respect to the undeformed aggregate effective viscosity. The models suggest that this maximum weakening occurs at viscosity contrasts of two orders of magnitude. With the matrix‐inclusion distributions considered here, the resulting system of equations becomes ill‐posed at higher viscosity contrasts. Further improvement on the stability of linear solvers is necessary to confirm the latter observation from a numerical point of view.

In aggregates with strong inclusions, a linear fabric develops at low viscosity contrasts, and the inclusions remain largely undeformed at moderate‐to‐large viscosity contrasts. The strength of the aggregate remains roughly constant even at high strain.

The anisotropic viscosity of two‐phase aggregates with weak inclusions, as well as aggregates with inclusions slightly stiffer than the surrounding matrix, is in good agreement with the solution of the DEM using an average shape and orientation of the fabric. Quantification of the anisotropic viscous tensor is of paramount importance for large scale geodynamic simulations since numerical limitations do not allow for a direct representation of multi‐phase aggregates.

The fabric shape and orientation of weak laminar fabrics is approximately proportional to bulk deformation in a linear manner, while high‐order polynomials are needed to approximate the development of L‐type fabrics. A combination of this parameterization with the DEM might be used to retrieve the anisotropic viscosity of the aggregate. Future work will implement this methodology to obtain the viscous tensor in modern geodynamic codes to explore the effects of viscous anisotropy on global mantle convection patterns and other large scale geodynamic processes.

## Supporting information

Supporting Information S1

Movie S1

Movie S2

Movie S3

## Figures and Tables

**Figure A1:**
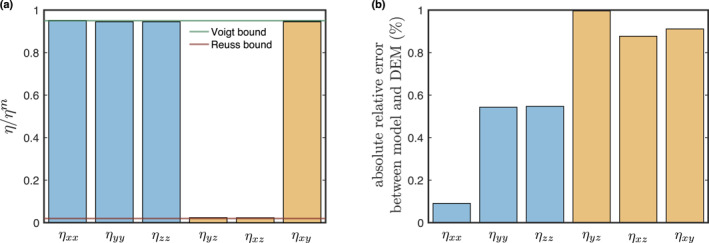
(a) Normalized components of the anisotropic viscous tensor and the Voigt and Reuss bounds. b) absolute relative error between the anisotropic viscous tensor recovered from the 3D models and the solution of the DEM equation. The matrix is 10^3^ more viscous than the weak layer.

**Figure B1:**
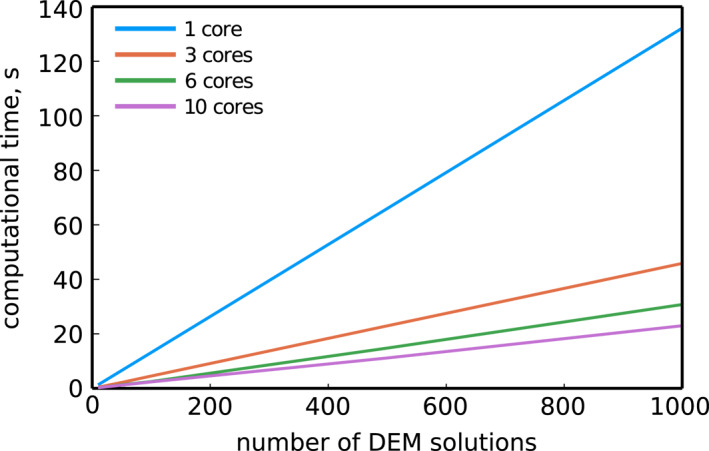
Computational cost of differential effective medium (DEM) solution for different numbers of threads. The volume fraction in this test is *ϕ* = 30% with Δ*ϕ* = 5 ⋅ 10^−3^. The Green interaction tensor is numerically integrated by discretizing the integration space in 100 nodes in both dimensions. Parallelization is done at the coarsest level and all the calculations were run on an AMD Ryzen 5 3600.

## Data Availability

The model output data shown in Movies S1, S2 and S3 and post‐processing script are publicly available (https://doi.org/10.6084/m9.figshare.14431460.v1).

## Appendix A Ideally Layered Two‐Phase Aggregate

The model set up consist of a weak horizontal layer of unit length in the *x* and *y* directions and thickness 0.05, embedded in the center of a unit length cubic matrix. Two rigid plates of thickness 0.08 are added to the top and bottom faces of the matrix. The viscosity of the matrix and layer is *η* ^*m*^ = 10^3^ and *η* ^*i*^ = 1, respectively. The shear components of the anisotropic viscosity are retrieved by imposing simple shear deformation on the model and solving the instantaneous flow. Unitary velocity ***v*** ^top^ = (1, 0, 0) and ***v*** ^bot^ = (−1, 0, 0) are imposed in the upper and lower rigid plates, respectively, while the vertical faces of the domain are periodic. The effective viscosity of the model corresponds to the shear anisotropic viscosity *η*_*xz*_. The *η*_*yz*_ and *η*_*xy*_ viscosities are obtained by rotating the weak layer and strong matrix along the *X* and *Y* axis, respectively, before solving the Stokes equation. The normal anisotropic viscosity components are obtained by imposing pure shear deformation on the aggregate. In this case, unitary velocity ***v*** ^top^ = (0, 0, −1) and ***v*** ^right^ = (1, 0, 0) are imposed in the upper and right faces of the domain, respectively, while free‐slip boundary condition is imposed in the remaining faces. The effective viscosity of the model corresponds to the normal component of the anisotropic tensor *η*_*xx*_. As in the simple shear experiments, the normal components *η*_*yy*_ and *η*_*zz*_ are obtained by rotating the weak layer and strong matrix along the x‐ and *y*‐axis, respectively, and solving the instantaneous flow. For the pure shear experiments, the rigid plates are removed.

The left‐hand‐side panel of Figure A1 shows a comparison between the normalized anisotropic viscosity obtained from the 3D model and the Voigt and Reuss bounds. As expected for a composite with a pseudo‐infinite weak layer, the normal viscosity components and *η*_*xy*_ closely follow the Voigt limit, while *η*_*yz*_ and *η*_*xz*_ match the Reuss bound (Figure A1a). In this particular case where the geometry of the weak layer is perfectly determined, the anisotropic viscosity of the 3D model is extremely well predicted by the DEM (Figure A1b), with relative errors below 1%. The mismatch between the 3D models and the DEM is because the DEM can not be solved for a perfectly layered composite. Here, we solved the DEM considering an inclusion with semi‐axes *a*_1_ = *a*_2_ = 10^3^ and *a*_3_ = 0.05.

## Appendix B Differential Effective Method and Numerical Implementation

We use a Julia package (de Montserrat, 2021) to calculate the anisotropic tensor by solving Equation 5. The computational cost of solving Equation 5 is heavily dominated by the integration of the Green interaction fourth order tensor, Equation 8. As a result, the calculation of the viscous tensor for a single aggregate is on the order of seconds for few computations (Figure B1) and increases linearly with the number of calculations. The calculation of the viscous tensor can be easily parallelized at the coarsest level over several CPU (Figure B1). However, solving the DEM on‐the‐fly remains not viable even after scaling to several hundreds of CPUs, given that the viscosity tensor is easily required to be computed at millions‐to‐billions points. The Green interaction tensor is integrated using a trapezoidal rule over two dimensions. Alternatively, M. Qu et al. (2016) employed different combinations of optimal integration quadratures. However, this approach is considerably outperformed by our implementation since less integration points are required to obtain an accurate solution within the range of inclusion shapes found in our models. As a computationally viable alternative, one can build a database of viscous tensors solving Equation 5 for a given range of physical parameters.

## References

[jgrb55256-bib-0001] Backus, G. E. (1962). Long‐wave elastic anisotropy produced by horizontal layering. Journal of Geophysical Research, 67(11), 4427–4440. 10.1029/jz067i011p04427

[jgrb55256-bib-0002] Ballmer, M. D. , Houser, C. , Hernlund, J. W. , Wentzcovitch, R. M. , & Hirose, K. (2017). Persistence of strong silica‐enriched domains in the earth's lower mantle. Nature Geoscience, 10(3), 236–240. 10.1038/ngeo2898

[jgrb55256-bib-0003] Becker, T. W. , Kellogg, J. B. , Ekström, G. , & O'Connell, R. J. (2003). Comparison of azimuthal seismic anisotropy from surface waves and finite strain from global mantle‐circulation models. Geophysical Journal International, 155(2), 696–714. 10.1046/j.1365-246x.2003.02085.x

[jgrb55256-bib-0004] Boucher, S. (1976). Modules effectifs de matériaux composite quasi homogénes et quasi isotropes, constitués d'une matrice élastique et d'inclusions élastiques. ii cas des concentrations finies en inclusions. Revue de Métallurgie, 22, 31–36.

[jgrb55256-bib-0005] Caricchi, L. , Pommier, A. , Pistone, M. , Castro, J. , Burgisser, A. , & Perugini, D. (2011). Strain‐induced magma degassing: Insights from simple‐shear experiments on bubble bearing melts. Bulletin of Volcanology, 73, 1245–1257. 10.1007/s00445-011-0471-2

[jgrb55256-bib-0006] Ceriani, S. , Mancktelow, N. S. , & Pennacchioni, G. (2003). Analogue modelling of the influence of shape and particle/matrix interface lubrication on the rotational behaviour of rigid particles in simple shear. Journal of Structural Geology, 25(12), 2005–2021. 10.1016/s0191-8141(03)00098-1

[jgrb55256-bib-0007] Christensen, U. R. (1987). Some geodynamical effects of anisotropic viscosity. Geophysical Journal International, 91(3), 711–736. 10.1111/j.1365-246x.1987.tb01666.x

[jgrb55256-bib-0008] Dabrowski, M. , Schmid, D. , & Podladchikov, Y. (2012). A two‐phase composite in simple shear: Effective mechanical anisotropy development and localization potential. Journal of Geophysical Research: Solid Earth, 117(B8). 10.1029/2012jb009183

[jgrb55256-bib-0009] De Coninck, A. , De Baets, B. , Kourounis, D. , Verbosio, F. , Schenk, O. , Maenhout, S. , & Fostier, J. (2016). Needles: Toward large‐scale genomic prediction with marker‐by‐environment interaction. Genetics, 203(1), 543–555. 10.1534/genetics.115.179887 26936924 PMC4858798

[jgrb55256-bib-0010] de Montserrat, A. (2021). Albert‐de‐Montserrat/dem: Dem. Zenodo. 10.5281/zenodo.4696200

[jgrb55256-bib-0011] Di Toro, G. , & Pennacchioni, G. (2004). Superheated friction‐induced melts in zoned pseudotachylytes within the adamello tonalites (Italian Southern Alps). Journal of Structural Geology, 26, 1783–1801. 10.1016/j.jsg.2004.03.001

[jgrb55256-bib-0012] Eshelby, J. D. (1957). The determination of the elastic field of an ellipsoidal inclusion, and related problems. Proceedings of the Royal Society of London ‐ Series A: Mathematical and Physical Sciences, 241(1226), 376–396.

[jgrb55256-bib-0013] Eshelby, J. D. (1959). The elastic field outside an ellipsoidal inclusion. Proceedings of the Royal Society of London ‐ Series A: Mathematical and Physical Sciences, 252(1271), 561–569.

[jgrb55256-bib-0014] Faccenda, M. , Ferreira, A. M. , Tisato, N. , Lithgow‐Bertelloni, C. , Stixrude, L. , & Pennacchioni, G. (2019). Extrinsic elastic anisotropy in a compositionally heterogeneous Earth's mantle. Journal of Geophysical Research: Solid Earth, 124(2), 1671–1687. 10.1029/2018jb016482 31008001 PMC6472509

[jgrb55256-bib-0015] Gee, L. S. , & Jordan, T. H. (1988). Polarization anisotropy and fine‐scale structure of the eurasian upper mantle. Geophysical Research Letters, 15(8), 824–827. 10.1029/gl015i008p00824

[jgrb55256-bib-0016] Gerya, T. (2019). Introduction to numerical geodynamic modelling. Cambridge University Press.

[jgrb55256-bib-0017] Gerya, T. V. , & Yuen, D. A. (2003). Characteristics‐based marker‐in‐cell method with conservative finite‐differences schemes for modeling geological flows with strongly variable transport properties. Physics of the Earth and Planetary Interiors, 140(4), 293–318. 10.1016/j.pepi.2003.09.006

[jgrb55256-bib-0018] Girard, J. , Amulele, G. , Farla, R. , Mohiuddin, A. , & Karato, S.‐i. (2016). Shear deformation of bridgmanite and magnesiowüstite aggregates at lower mantle conditions. Science, 351(6269), 144–147. 10.1126/science.aad3113 26721681

[jgrb55256-bib-0019] Handy, M. R. (1990). The solid‐state flow of polymineralic rocks. Journal of Geophysical Research: Solid Earth, 95(B6), 8647–8661. 10.1029/jb095ib06p08647

[jgrb55256-bib-0020] Hansen, L. , Zimmerman, M. , & Kohlstedt, D. (2012). Laboratory measurements of the viscous anisotropy of olivine aggregates. Nature, 492(7429), 415–418. 10.1038/nature11671 23257885

[jgrb55256-bib-0021] Honda, S. (1986). Strong anisotropic flow in a finely layered asthenosphere. Geophysical Research Letters, 13(13), 1454–1457. 10.1029/gl013i013p01454

[jgrb55256-bib-0022] Jiang, D. (2012). A general approach for modeling the motion of rigid and deformable ellipsoids in ductile flows. Computers & Geosciences, 38(1), 52–61. 10.1016/j.cageo.2011.05.002

[jgrb55256-bib-0023] Jiang, D. (2014). Structural geology meets micromechanics: A self‐consistent model for the multiscale deformation and fabric development in earth's ductile lithosphere. Journal of Structural Geology, 68, 247–272. 10.1016/j.jsg.2014.05.020

[jgrb55256-bib-0024] Jiang, D. , & Bhandari, A. (2018). Pressure variations among rheologically heterogeneous elements in earth's lithosphere: A micromechanics investigation. Earth and Planetary Science Letters, 498, 397–407. 10.1016/j.epsl.2018.07.010

[jgrb55256-bib-0025] Kaminski, E. , Ribe, N. M. , & Browaeys, J. T. (2004). D‐rex, a program for calculation of seismic anisotropy due to crystal lattice preferred orientation in the convective upper mantle. Geophysical Journal International, 158(2), 744–752. 10.1111/j.1365-246x.2004.02308.x

[jgrb55256-bib-0026] Kilian, R. , Heilbronner, R. , & Stünitz, H. (2011). Quartz grain size reduction in a granitoid rock and the transition from dislocation to diffusion creep. Journal of Structural Geology, 33(8), 1265–1284. 10.1016/j.jsg.2011.05.004

[jgrb55256-bib-0027] Király, Á. , Conrad, C. P. , & Hansen, L. N. (2020). Evolving viscous anisotropy in the upper mantle and its geodynamic implications. Geochemistry, Geophysics, Geosystems, 21(10), e2020GC009159. 10.1029/2020gc009159

[jgrb55256-bib-0028] Kocher, T. , Schmalholz, S. M. , & Mancktelow, N. S. (2006). Impact of mechanical anisotropy and power‐law rheology on single layer folding. Tectonophysics, 421(1–2), 71–87. 10.1016/j.tecto.2006.04.014

[jgrb55256-bib-0029] Komar, P. D. (1972). Mechanical interactions of phenocrysts and flow differentiation of igneous dikes and sills. Geological Society of America Bulletin, 83(4), 973–988. 10.1130/0016-7606(1972)83[973:miopaf]2.0.co;2

[jgrb55256-bib-0030] Kourounis, D. , Fuchs, A. , & Schenk, O. (2018). Towards the next generation of multiperiod optimal power flow solvers. IEEE Transactions on Power Systems, 33(99), 1–4014. 10.1109/TPWRS.2017.2789187

[jgrb55256-bib-0031] Lebensohn, R. , Turner, P. , Signorelli, J. , Canova, G. , & Tomé, C. (1998). Calculation of intergranular stresses based on a large‐strain viscoplastic self‐consistent polycrystal model. Modelling and Simulation in Materials Science and Engineering, 6(4), 447–465. 10.1088/0965-0393/6/4/011

[jgrb55256-bib-0032] Lev, E. , & Hager, B. H. (2008). Rayleigh–taylor instabilities with anisotropic lithospheric viscosity. Geophysical Journal International, 173(3), 806–814. 10.1111/j.1365-246x.2008.03731.x

[jgrb55256-bib-0033] Mainprice, D. (1997). Modelling the anisotropic seismic properties of partially molten rocks found at mid‐ocean ridges. Tectonophysics, 279(1–4), 161–179. 10.1016/s0040-1951(97)00122-4

[jgrb55256-bib-0034] Mancktelow, N. S. , Arbaret, L. , & Pennacchioni, G. (2002). Experimental observations on the effect of interface slip on rotation and stabilisation of rigid particles in simple shear and a comparison with natural mylonites. Journal of Structural Geology, 24(3), 567–585. 10.1016/s0191-8141(01)00084-0

[jgrb55256-bib-0035] May, D. A. , Brown, J. , & Le Pourhiet, L. (2015). A scalable, matrix‐free multigrid preconditioner for finite element discretizations of heterogeneous stokes flow. Computer Methods in Applied Mechanics and Engineering, 290, 496–523. 10.1016/j.cma.2015.03.014

[jgrb55256-bib-0036] McKenzie, D. (1979). Finite deformation during fluid flow. Geophysical Journal International, 58(3), 689–715. 10.1111/j.1365-246x.1979.tb04803.x

[jgrb55256-bib-0037] McLaughlin, R. (1977). A study of the differential scheme for composite materials. International Journal of Engineering Science, 15(4), 237–244. 10.1016/0020-7225(77)90058-1

[jgrb55256-bib-0038] Moulas, E. , Burg, J.‐P. , & Podladchikov, Y. (2014). Stress field associated with elliptical inclusions in a deforming matrix: Mathematical model and implications for tectonic overpressure in the lithosphere. Tectonophysics, 631, 37–49. 10.1016/j.tecto.2014.05.004

[jgrb55256-bib-0039] Mühlhaus, H.‐B. , Dufour, F. , Moresi, L. , & Hobbs, B. (2002). A director theory for visco‐elastic folding instabilities in multilayered rock. International Journal of Solids and Structures, 39(13–14), 3675–3691. 10.1016/s0020-7683(02)00175-0

[jgrb55256-bib-0040] Mura, T. (1987). Micromechanics of deffects in solids. Springer.

[jgrb55256-bib-0041] Nemat‐Nasser, S. , & Hori, M. (2013). Micromechanics: Overall properties of heterogeneous materials (Vol. 37). Elsevier.

[jgrb55256-bib-0042] Passchier, C. W. , & Sokoutis, D. (1993). Experimental modelling of mantled porphyroclasts. Journal of Structural Geology, 15(7), 895–909. 10.1016/0191-8141(93)90183-b

[jgrb55256-bib-0043] Perry‐Houts, J. , & Karlstrom, L. (2019). Anisotropic viscosity and time‐evolving lithospheric instabilities due to aligned igneous intrusions. Geophysical Journal International, 216(2), 794–802. 10.1093/gji/ggy466

[jgrb55256-bib-0044] Qu, J. , & Cherkaoui, M. (2006). Fundamentals of micromechanics of solids (Vol. 735). Wiley Online Library.

[jgrb55256-bib-0045] Qu, M. , Jiang, D. , & Lu, L. X. (2016). An optimal scheme for numerical evaluation of eshelby tensors and its implementation in a matlab package for simulating the motion of viscous ellipsoids in slow flows. Computers & Geosciences, 96, 98–108. 10.1016/j.cageo.2016.08.005

[jgrb55256-bib-0046] Ranalli, G. (1995). Rheology of the earth. Springer Science & Business Media.

[jgrb55256-bib-0047] Roscoe, R. (1952). The viscosity of suspensions of rigid spheres. British Journal of Applied Physics, 3(8), 267–269. 10.1088/0508-3443/3/8/306

[jgrb55256-bib-0048] Skemer, P. , Warren, J. M. , Kelemen, P. B. , & Hirth, G. (2010). Microstructural and rheological evolution of a mantle shear zone. Journal of Petrology, 51(1–2), 43–53. 10.1093/petrology/egp057

[jgrb55256-bib-0049] Stixrude, L. , & Lithgow‐Bertelloni, C. (2012). Geophysics of chemical heterogeneity in the mantle. Annual Review of Earth and Planetary Sciences, 40, 569–595. 10.1146/annurev.earth.36.031207.124244

[jgrb55256-bib-0050] Thielmann, M. , Golabek, G. J. , & Marquardt, H. (2020). Ferropericlase control of lower mantle rheology: Impact of phase morphology. Geochemistry, Geophysics, Geosystems, 21(2), e2019GC008688. 10.1029/2019GC008688

[jgrb55256-bib-0051] Verbosio, F. , Coninck, A. D. , Kourounis, D. , & Schenk, O. (2017). Enhancing the scalability of selected inversion factorization algorithms in genomic prediction. Journal of Computational Science, 22(Supplement C), 99–108. 10.1016/j.jocs.2017.08.013

[jgrb55256-bib-0052] Yamazaki, D. , Yoshino, T. , Matsuzaki, T. , Katsura, T. , & Yoneda, A. (2009). Texture of (Mg, Fe) SiO_3_ perovskite and ferro‐periclase aggregate: Implications for rheology of the lower mantle. Physics of the Earth and Planetary Interiors, 174(1–4), 138–144. 10.1016/j.pepi.2008.11.002

[jgrb55256-bib-0053] Yang, R. , Jiang, D. , & Lu, L. X. (2019). Constrictional strain and linear fabrics as a result of deformation partitioning: A multiscale modeling investigation and tectonic significance. Tectonics, 38(8), 2829–2849. 10.1029/2019tc005490

