# Supplementary material for: Extrinsic Anisotropy of Two‐Phase Newtonian Aggregates: Fabric Characterization and Parameterization
Source: J Geophys Res Solid Earth. 2021 Oct 29;126(11):e2021JB022232. doi: 10.1029/2021JB022232 (PMC9285778; doi:10.1029/2021JB022232)
Supplement: Supplementary file 1 — Supporting Information S1 [file JGRB-126-0-s003.docx]

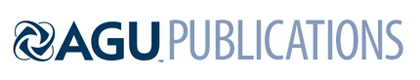


*[JGR – Solid Earth]*

Supporting Information for

**Extrinsic anisotropy of two-phase Newtonian aggregates: Fabric characterization and parameterization**

Albert de Montserrat^1^, Manuele Faccenda^1^, Giorgio Pennacchioni^1^

*^1^ Dipartimento di Geoscienze, Università di Padova, via Gradenigo 6, Padova, Italy*

**Supporting Information: Movies (files uploaded separately)*.***

Captions for Movies S1 to S3

**Movie S1.** Development of foliated fabric in two-phase aggregate in simple shear for inclusions $10$ times weaker than the matrix and volume fraction 0.2. Bottom panels show the evolution of the normalized viscosity and strain partitioning. Initial setup is shown in Figure S1.h.

**Movie S2.** Development of foliated fabric in two-phase aggregate in simple shear for inclusions ${10}^{3}$ times weaker than the matrix and volume fraction 0.2. Bottom panels show the evolution of the normalized viscosity and strain partitioning. Initial setup is shown in Figure S1.b.

**Movie S3.** Development of lineated fabric in two-phase aggregate in simple shear for inclusions $10$ times stronger than the matrix and volume fraction 0.2. Bottom panels show the evolution of the normalized viscosity and strain partitioning. Initial setup is shown in Figure S2.a.

**Supporting Information: Figures**

| **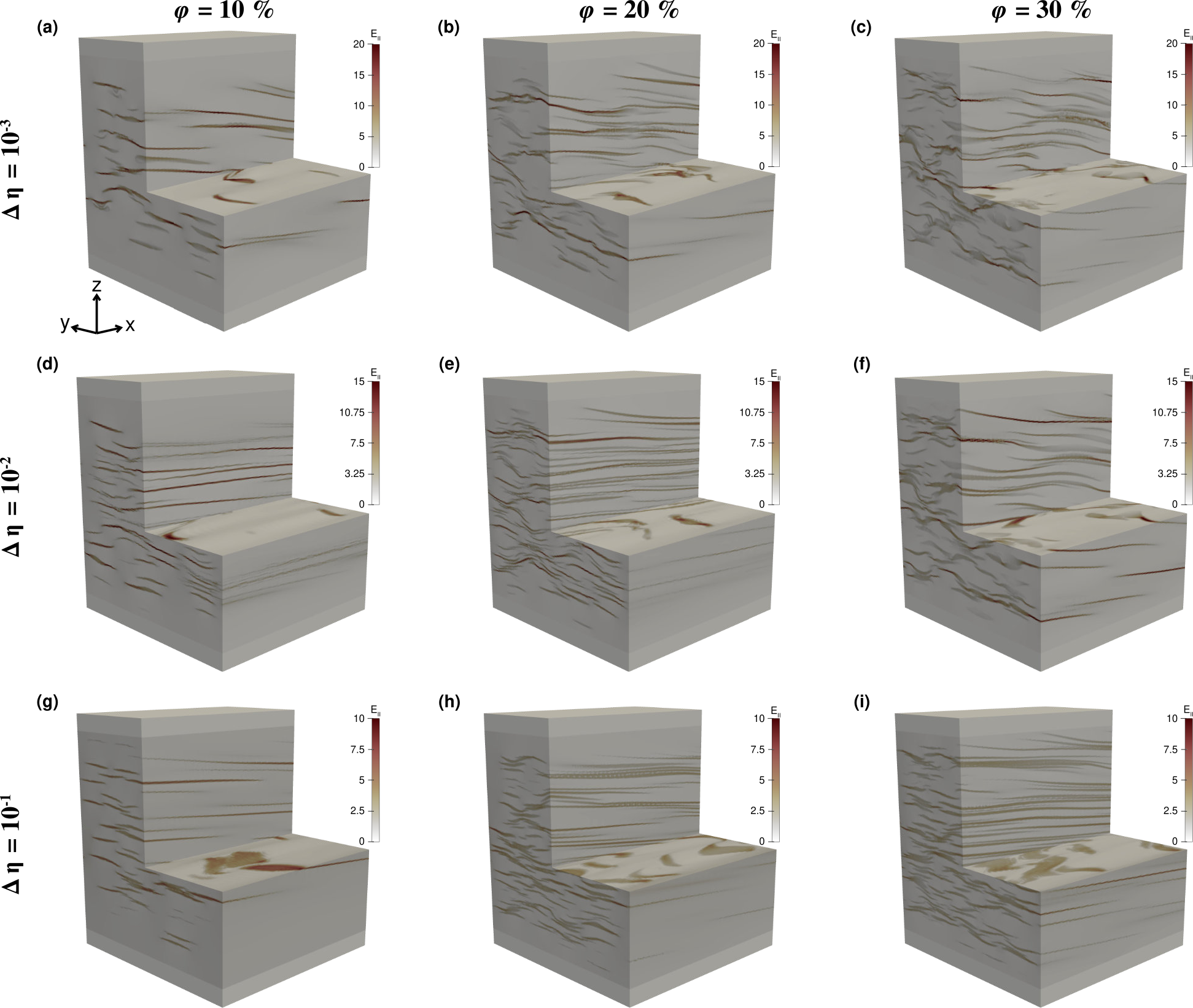** |
| --- |
| **Figure S1.** Second invariant of the finite strain tensor (E_II_) at $\gamma=6$ for two-phase aggregates with weak inclusions. |

| 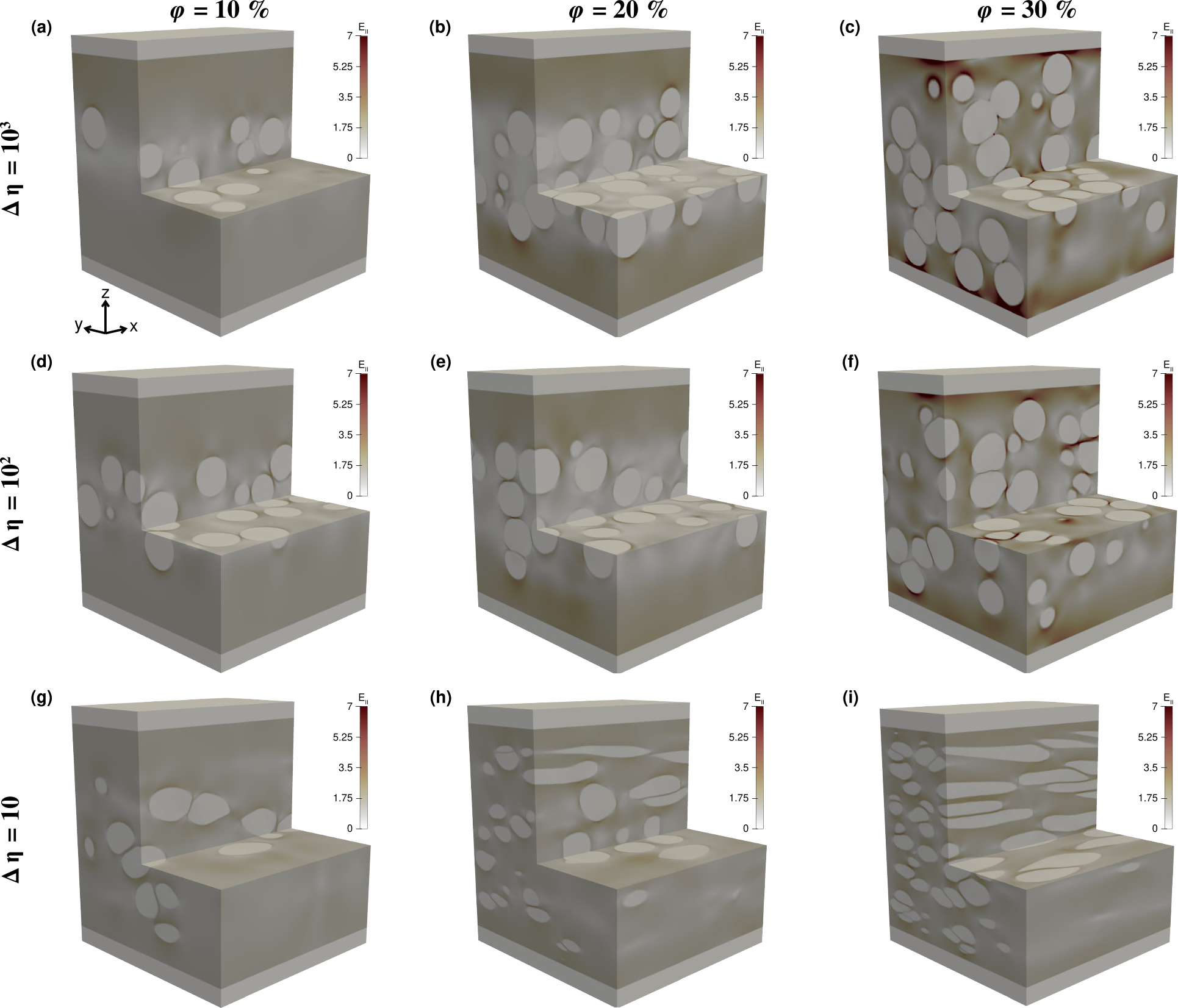 |
| --- |
| **Figure S2.** Second invariant of the finite strain tensor (E_II_) at $\gamma=16.4$ for two-phase aggregates with strong inclusions. Shear deformation in panel **i** is $\gamma=6$ due to lack of convergence of the solution. |

| 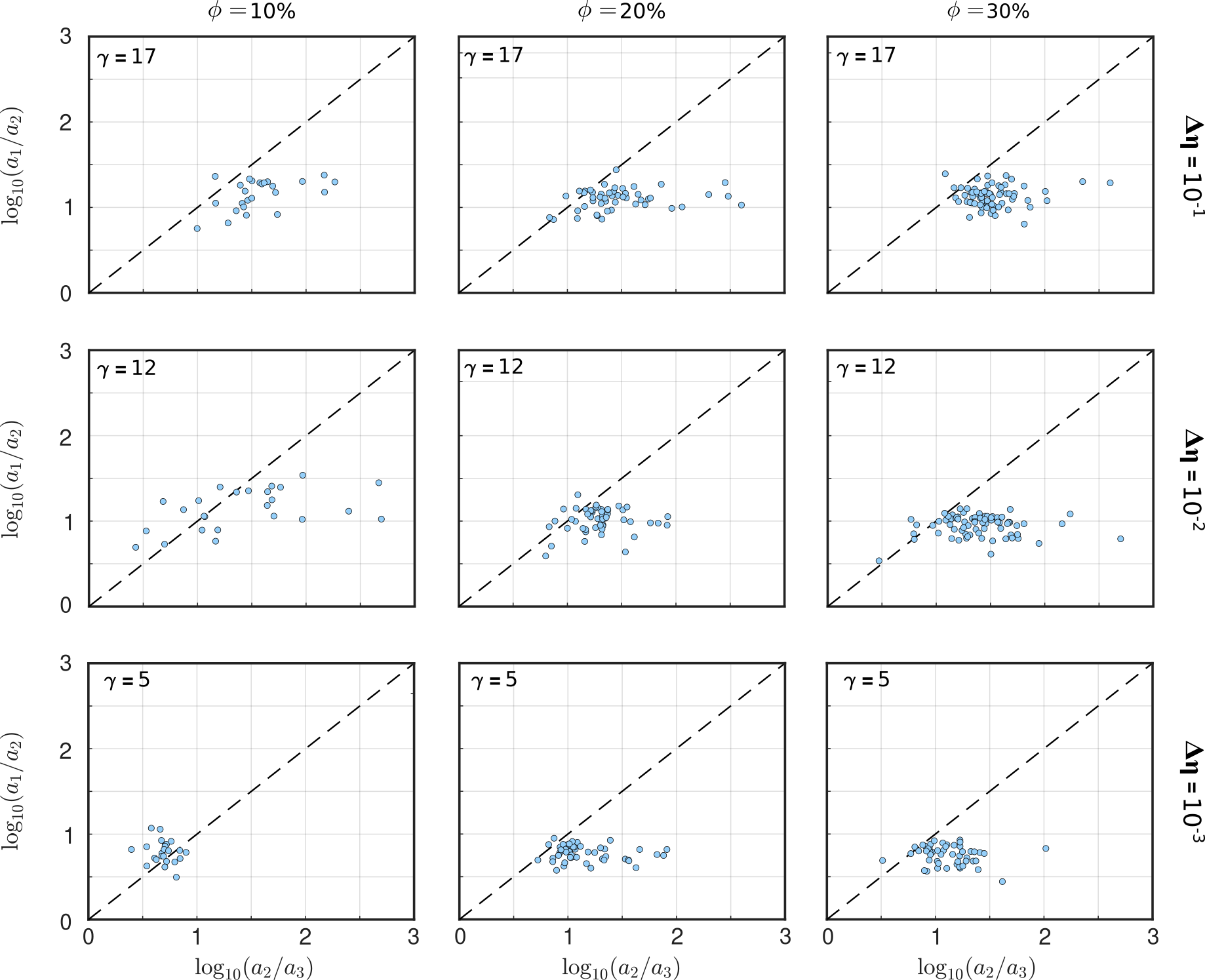 |
| --- |
| **Figure S3.** Flinn diagrams of two-phase aggregates with weak inclusions under simple shear boundary conditions. |

| 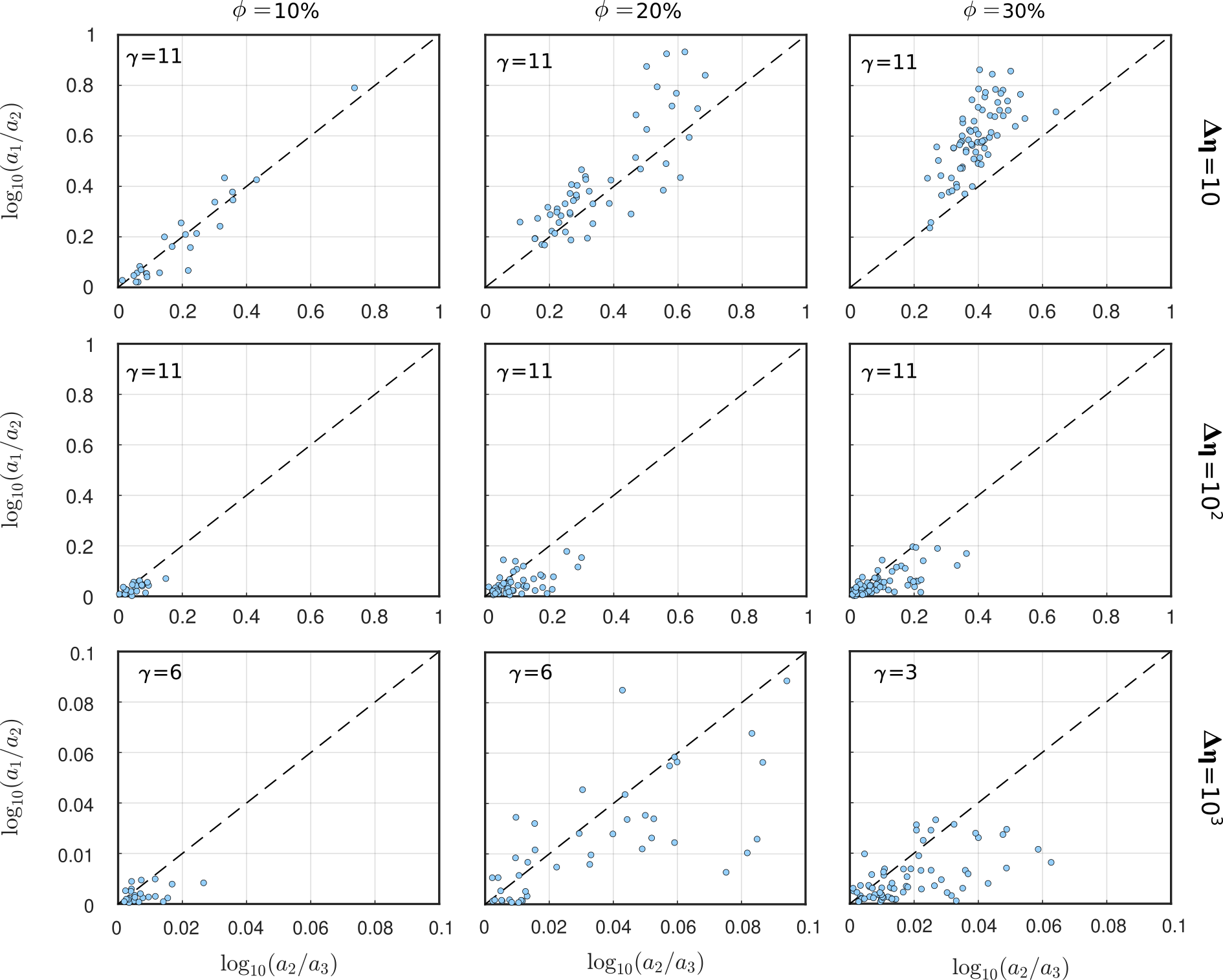 |
| --- |
| **Figure S4.** Flinn diagrams of two-phase aggregates with strong inclusions under simple shear boundary conditions. |

| 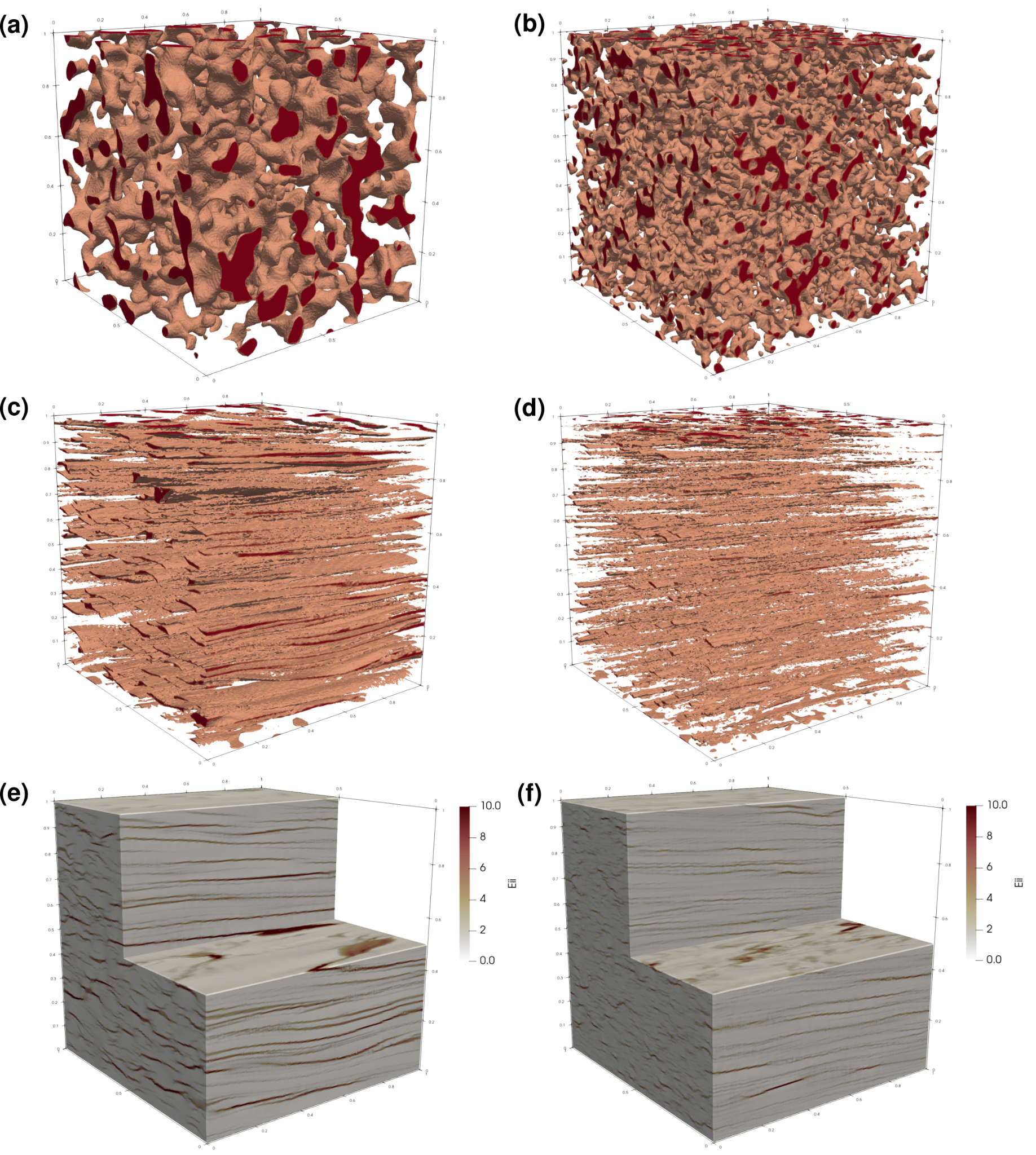 |
| --- |
| **Figure S5.** **(a)** and **(b),** initial aggregate morphology. The inclusion phase has been generated using the statistical approach in Thielmann et al. (2020) that yields a heterogeneous random media which is statistically isotropic. **(c)** and **(d)**, aggregate fabric. **(e)** and **(f)**, second invariant of the strain rate tensor (E_II_). The volume fraction of the inclusion phase is 20%. Panels **(c), (d)**, **(e),** and **(f)** correspond to the model with $\Delta\eta={10}^{-2}$, at $\gamma=15$. |
